# Quantifying the spatio-temporal image degradation under motion blur in fluorescence microscopy

**DOI:** 10.1016/j.bpr.2026.100290

**Published:** 2026-09-23

**Authors:** Serafim Korovin, Kutay Ugurlu, Dylan Kalisvaart, Manon Kok, Rainer Heintzmann, Kirti Prakash, Carlas S. Smith

**Affiliations:** 1 Delft Center for Systems and Control, Department of Mechanical Engineering, Delft University of Technology, Delft 2628, the Netherlands; 2 Leibniz Institute of Photonic Technology, Jena, Germany; 3 Institute of Physical Chemistry and Abbe Center of Photonics, Friedrich Schiller University Jena, Jena, Germany; 4 School of Computing and Information Science, Anglia Ruskin University, East Road, Cambridge, UK

## Abstract

The spatial resolution of optical imaging systems is fundamentally restricted by the diffraction limit. However, in widefield live-cell microscopy, the achievable resolution is further constrained by specimen motion, which indicates the existence of a fundamental spatio-temporal resolution trade-off between signal accumulation during full frame integration and the resulting motion blur. To improve the fidelity with which moving objects can be imaged, a quantitative understanding of this spatio-temporal trade-off is necessary. Here, we present a systematic analysis of motion-induced resolution dynamics measured with the spectral signal-to-noise ratio (SSNR). We developed a simulation framework that models the image formation of objects undergoing arbitrary motion to evaluate the degradation of the spatial resolution under translational, diffusive, and rotational dynamics. Our results demonstrate that for translating objects, the spatial resolution is anisotropically reduced as a function of the orientation of the object relative to the motion vector, leading to the SSNR degrading by up to 50% and the resolution by up to 40% for a 90° change in the motion direction. This motion-blur-induced spectral degradation was further observed on simulated points under Brownian motion and dynamic *Drosophila* data. Moreover, we show that for rotational motion, conventional radially averaged metrics such as the Fourier ring correlation cannot quantify the effects of angular blur, which the SSNR quantifies accurately. These findings underscore the necessity of an object-oriented imaging approach, in which acquisition parameters such as exposure time are tuned to specific biological spatio-temporal characteristics to optimize the trade-off between motion blur and spatial fidelity.

## Why it matters

Motion is an unavoidable feature of living systems and can substantially limit the spatial resolution achievable in microscopy. Although longer exposure times improve signal collection, they also increase motion blur, creating an inherent trade-off between image quality and temporal dynamics. Here, we systematically quantify how different forms of motion affect recoverable spatial information using the spectral signal/noise ratio (SSNR). Our analysis reveals direction-dependent resolution losses during linear motion, measurable degradation due to diffusion, and the limitations of conventional resolution metrics for rotational motion. Along with these findings, we suggest a framework for selecting imaging conditions that preserve structural fidelity while capturing dynamic biological behavior.

## Introduction

Over the last two decades, developments in microscopy have largely been driven by efforts to overcome the Abbe diffraction limit. Modern fluorescence microscopy achieves high molecular specificity, selectivity, and chemical contrast by using fluorophores that target specific biological samples.^1^ With the introduction of super-resolution microscopy modalities, such as structured illumination microscopy (SIM),^2^^,^^3^^,^^4^^,^^5^ stimulated emission depletion (STED),^6^^,^^7^^,^^8^^,^^9^ and single-molecule localization microscopy (SMLM),^10^^,^^11^^,^^12^ spatial resolution has successfully been extended down to the nanometer scale. Yet these spatial improvements fundamentally come at the cost of temporal trade-offs.^13^ Consequently, maximizing spatial resolving power fundamentally necessitates prolonged acquisition times.^1^^,^^14^^,^^15^ While this resolution trade-off is explicitly defined in super-resolution imaging, an analogous trade-off also can be observed as resolution-degrading motion blur in the widefield regime.

This trade-off introduces a severe bottleneck when imaging dynamic live-cell environments.^1^^,^^16^^,^^17^ When the standard assumption of a static specimen is violated, the continuous-time emission signal is smeared across the detector during the extended integration time, irreversibly constraining the maximum recoverable spatial frequencies. This effect, known as motion blur, fundamentally degrades image fidelity. The impact of motion blur becomes particularly problematic in fast-moving biological systems. For instance, highly dynamic processes, such as bacterial motility, frequently exhibit directional velocities reaching up to 50 nm ms^−1^ ^18^(Table S1). Assuming a standard 40 ms acquisition time , the target structure undergoes displacement of 2 μm within a single acquired frame. This corresponds to the signal integrated over more than 30 pixels (for a 60 nm projected pixel size^19^) and more than 19 PSF standard deviations (for a Gaussian-shaped point spread function [PSF] with a standard deviation of 103.5 nm) in the images obtained from the camera. At the nanoscale, this substantial morphological distortion renders the resulting motion blur virtually indistinguishable from the object’s genuine biological structure.

To mitigate this spatio-temporal degradation, various hardware solutions have been developed. On the instrumentation front, extremely fast SIM systems have been developed that generate illumination patterns by switching between different fibers.^20^ As a result, acquisition times can be improved up to 408 frames per second. On the detection side, continued progress in scientific complementary metal-oxide-semiconductor (sCMOS) cameras and single-photon avalanche diode (SPAD) arrays has significantly advanced the achievable imaging speeds, with modern sCMOS detectors supporting kHz-level frame acquisitions^21^ and specialized SPAD architectures such as SwissSPAD^22^^,^^23^ enabling frame rates of hundreds of kHz. These advancements enable kHz-level imaging with effectively zero readout noise and sub-nanosecond timing, thereby reducing required exposure times.

In addition to these hardware solutions, computational solutions have also been developed to reduce the spatio-temporal information loss. Particularly over the last few years, we have seen neural networks (NNs) transform low-resolution measurements into astonishingly super-resolved reconstructions of the cell.^24^^,^^25^^,^^26^^,^^27^^,^^28^^,^^29^ Deep-learning frameworks such as CARE and DBlink have emerged to reconstruct super-resolved dynamics directly from low-signal/noise ratio (SNR) data and motion-blurred SMLM data, respectively, effectively bypassing the need for long temporal baselines.^30^^,^^31^ Alternatively, BNP-TRACK mitigates the effect of motion blur in super-resolved reconstructions by combining single-particle tracking with super-resolved imaging in a single Bayesian framework.^32^ By estimating the joint posterior of the number of emitters, the emitter positions, and their tracks, BNP-TRACK reaches a localization precision of approximately 50 nm on dynamic data.

However, despite these advanced mitigation strategies, a quantitative understanding of how motion physically erodes the spatial frequency spectrum remains incomplete. While algorithms such as DBlink effectively circumvent motion blur, thereby seemingly conjuring more information from less data, their black-box architectures obscure whether the recovered high-frequency details originate directly from the raw measurements or from learned priors embedded during network training.^30^ To research the physical integrity with which these methods obtain a reconstruction, we need a quantitative and fundamental understanding of how motion blur affects spatial image frequencies. Additionally, to advance beyond empirical correction toward true object-oriented, adaptive imaging, it is critical to explicitly quantify the origin and degradation of spatio-temporal information.^33^

Therefore, in this work, we systematically develop a quantitative framework to model how object kinematics fundamentally erode spatial resolution in widefield fluorescence microscopy. By simulating benchmark targets undergoing linear, rotational, and diffusive planar motion and analyzing dynamic light-sheet fluorescence data, we evaluate the anisotropic redistribution of spatial frequencies across varying exposure durations using the spectral signal/noise ratio (SSNR) metric. Originally developed in cryo-electron microscopy to evaluate the SNR in Fourier space,^34^ it was adapted in SIM systems^2^^,^^35^ to evaluate the frequency-domain information content of the images. Crucially, because conventional radially averaged metrics such as Fourier ring correlation (FRC) are blind to the angular blur induced by rotation, we use an SSNR-based metric dynamically calibrated against background variance. Our choice of SSNR is also motivated by the fact that motion blur is observed in the spatial domain as a loss of fine structural detail. Since spatial-domain blurring corresponds to attenuation and redistribution of image information in the frequency domain, we expected motion-induced degradation to manifest as a frequency-dependent rescaling of the recoverable signal. Because the SSNR explicitly quantifies the SNR at each spatial frequency, it provides a natural framework for assessing how motion progressively suppresses information across the spectrum and for identifying the frequencies most affected by blur. Through this approach, we aim to establish rigorous mathematical boundaries for motion blur, identifying the precise points at which photon accumulation shifts from constructive to destructive, thereby providing practical guidelines for preserving structural fidelity in dynamic imaging environments.

The rest of the paper is structured as follows: materials and methods describes the simulation procedure developed to facilitate our investigation, defines the SSNR, and formalizes the SSNR-based frequency cutoff. The numerical simulation results are presented in results, where we demonstrate the impact of representative motions on the resolution of various object types. We conclude with a discussion and an outline for future work in conclusion.

## Materials and methods

### Image formation model

To quantitatively investigate how motion affects spatial resolution, we developed a simulator that models optical image formation for moving objects. We aimed to develop a time-dependent motion- and shape discretization of an object with high fidelity to its continuous representation and to simulate realistic camera measurements from it using a photon-counting process represented by Poisson shot noise. The simulation pipeline consists of five main stages, which are briefly summarized in this subsection. An exhaustive description of the simulation process can be found in Note S3. The parameters used throughout our simulation are described in Table S1.

We start by defining a continuous shape based on the intensity flux of the object and background flux. For this, we define a binary shape *S*(*x*,*y*,*t*) for continuous space-time coordinates (*x*,*y*,*t*), which takes the value 1 at space-time coordinates (*x*,*y*,*t*) if the object is present and 0 otherwise. This definition includes both object geometry and motion-induced position changes. Then, *S*(*x*,*y*,*t*) is scaled with a signal flux term *θ*_I_ (photons/(nm^2^·ms)), representing the object intensity. To obtain the continuous total flux (*x*,*y*,*t*) (photons/(nm^2^·ms)), a constant background flux *θ*_bg_ (photons/(nm^2^·ms)) is added to every space-time coordinate, which describes an expected intensity offset due to homogeneous background fluorescence. The model of *o*(*x*,*y*,*t*) is thus given by

(1) $$o\left(x,y,t\right)=\theta_{I}S\left(x,y,t\right)+\theta_{\mathrm{bg}}\text{.}$$

We then represent the continuous object flux on a finely discretized spatio-temporal mesh with voxel size *δV* = *δxδyδt*(nm^2^·ms). The continuous object flux *o*(*x*,*y*,*t*) is sampled at coordinates corresponding to the mesh pixel centers (*x*^(*i*)^,*y*^(*j*)^,*t*^(*k*)^) for respective pixels at indices *i*,*j* and discrete time index *k*. Additionally, *o*(*x*,*y*,*t*) is multiplied by the mesh voxel size *δV* to preserve the total photon flux within the spatio-temporal voxel. As a result, we arrive at the following definition:

(2) $$o_{i,j,k}=o\left(x^{\left(i\right)},y^{\left(j\right)},t^{\left(k\right)}\right)\delta V\text{.}$$

Next, we simulate a band-limited object representation on the image plane. To this end, the resulting discrete object *o*_*i*,*j*,*k*_ is convolved in space with the PSF of the simulated optical system. The PSF is represented as a Gaussian kernel with a standard deviation *σ* and a kernel support of *s* × *s* pixels. The Gaussian is a common choice for approximating the impulse response of optical systems, as it is both fast to compute and sufficiently accurate in 2D.^36^ Additionally, our simulation shows that the implementation is numerically band limited, with the spatial frequency at which the optical transfer function (OTF) amplitude drops below 10^–10^ × max(OTF) being 1/3 of the Nyquist frequency.

(3) $$I_{k}=o_{k}*\text{PSF}\left(\sigma ,s\right)\text{,}$$

where ∗ refers to the spatial convolution and *I*_*k*_ and *o*_*k*_ refer to the pixelated images at timestep *k*.

Finally, we simulate the camera readout process, which includes pixel discretization and photon counting during the exposure time, taken to be the inverse of the camera frame rate. We achieve that by binning the fine mesh pixels into camera pixels with factors (*C*_*x*_,*C*_*y*_) and binning each fine mesh frame with a factor *C*_*t*_. The resulting camera image ${\hat{I}}_{p,q,r}$ for respective camera pixels at indices *p*,*q* and frame index *r* is defined as follows:

(4) $${\hat{I}}_{p,q,r}=\sum_{i=0}^{C_{x}-1}\sum_{j=0}^{C_{y}-1}\sum_{k=0}^{C_{t}-1}I_{pC_{x}+i,qC_{y}+j,rC_{t}+k}\text{.}$$

To model the photon-counting process, a shot-noise realization ${\hat{I}}_{p,q,r}^{\text{noised}}$ is sampled from a Poisson distribution with the expected value of ${\hat{I}}_{p,q,r}$:

(5) $${\hat{I}}_{p,q,r}^{\text{noised}}∼\text{Pois}\left({\hat{I}}_{p,q,r}\right)\text{.}$$

### Estimation of SSNR and resolution

We quantify image resolution using the SSNR.^34^ The SSNR is computed directly from groups of noisy image realizations, all drawn from the same expected time-integrated image given by Equation 4, to accurately reflect experimental noise characteristics without relying on assumed noise models. We express SSNR as the ratio of the mean spectra of the images to the noise power computed as the variance of the spectra of the realizations. It scales linearly with exposure time, which makes it more convenient to evaluate the change in resolution with varying exposure times. To ensure isotropic evaluation, the resulting SSNR values are averaged over all angles, yielding a direction-independent resolution metric. The detailed mathematical formulation for the batch-wise SSNR computation and radial averaging is provided in the Note S4.

The SSNR decreases smoothly with spatial frequency rather than ending at a sharp boundary; thus, the curve does not contain an intrinsic “resolution point.” To extract a single resolution value from this curve, one must therefore define a cutoff marking where the remaining signal becomes indistinguishable from noise. Standard thresholds from other methods, such as FRC, are not suitable to define a threshold because they do not align with the SSNR value range. Instead, we define the resolution cutoff as the radial frequency at which the SSNR drops to the background SSNR level.^34^ We also measure the total SSNR in a frequency band by integrating the SSNR curve. The mathematical procedure to obtain this threshold is provided in Note S6.

## Results and discussion

To quantify the effect of exposure interval on image formation and resolution, we conduct numerical simulations that isolate the impact of motion. We consider three basic geometries subjected to distinct motion types to illustrate these effects: a line undergoing translation, a point under diffusive motion, and a Siemens star undergoing rotation. The line object offers a spectrum localized to one dimension, serving as a simplified model for elongated biological structures such as cytoskeletal filaments or membrane features.^37^ Diffusive point emitters represent biologically relevant scenarios encountered when imaging fluorescent sparsely labeled molecules undergoing stochastic motion,^38^ and the Siemens star, widely used for resolution assessment,^39^ provides rotational symmetry, which makes it particularly suitable for analyzing angular motion blur.^40^ Together, these geometries capture fundamental spatial characteristics common in fluorescence microscopy while remaining sufficiently simple to disentangle the effects of morphology from those of motion. More on the object formation and motion types can be found in Note S3.1. Furthermore, in Note S2, we derive the effects of translational and rotational motion for general object representations and demonstrate that the resulting spectral transformations are determined by the motion itself, independent of both the object morphology and the microscope PSF.

This section is structured as follows: we first set a baseline by reporting the SNR in the spatial domain for a static Siemens star and the SSNR for a static line. Next, we examine a rectangular object under translational motion and various orientations relative to the motion direction (Figure 3). We then analyze the point under Brownian motion (Figure 4) and the Siemens star under rotational motion (Figure 5), where spectral modification induced by motion manifests mainly in the angular blur. The results are reported based on the relevant evaluations from Table S2 for the quantification of Figure 2, Tables S4 and S5 provide numerical results for Figure 3, and Tables S6 and S7 quantify Figure 5.

### Static object imaging

In this section, we demonstrate and verify that the SNR increases with higher exposure time and intensity and decreases with a higher background (as background is not part of the signal). Figure 1 presents a static Siemens star with eight spokes limited to a radius of 3.6 μm imaged under varying exposure durations and background conditions. Figures 1a and b illustrate how the measurement is affected under varying background rates and exposure times, respectively.

**Figure 1:**
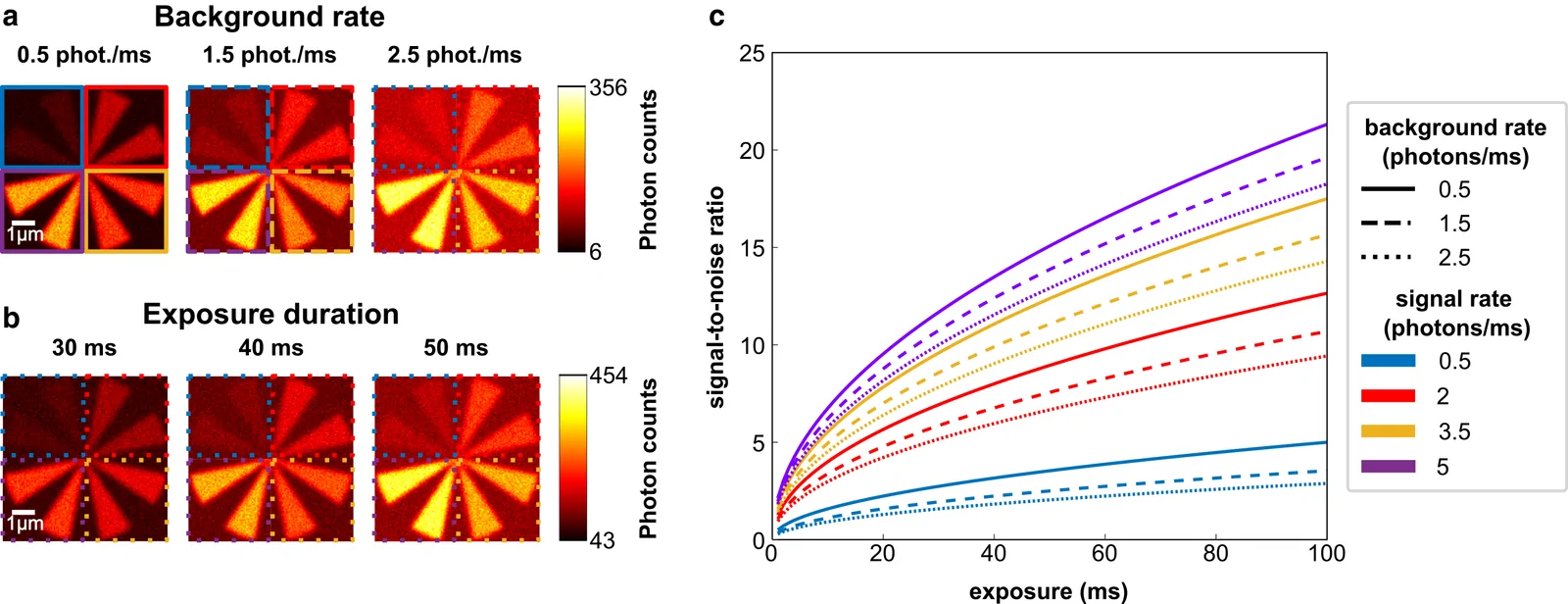
(a and b) A stationary Siemens star with varying (a) background rates (*λ*_bg_) imaged for 40 ms and (b) exposure times at a background rate of 2.5 photons/ms. Each image is divided into color-coded quadrants, where each quadrant encodes the corresponding signal rate (*λ*_I_ in photons/ms), and the line type encodes the background rate (in photons/ms), as described in the legend. (c) Simulated signal/noise ratios across a range of exposure times for varying signal and background rates.

Figure 1c shows the maximum SNR in each image computed using Equation S6. Exposure time increases as the square root of the expected SNR, while the SNR dependence on signal intensity is given by the factor $\lambda_{I}/\sqrt{\lambda_{I}+\lambda_{\text{bg}}}$. For example, raising the exposure time from 10 to 100 ms (with fixed signal and background) increases the SNR by a factor of $\sqrt{10}$. Moreover, increasing the signal rate from 0.5 to 5 for any fixed exposure time Δ*t* increases this factor from $0.50\sqrt{\Delta t}$ to $2.13\sqrt{\Delta t}$, resulting in a 4.26 times higher SNR (with a background rate of 0.50 photons). In contrast, increasing the background from a 0.5 to a 2.5 photon rate lowers the SNR from $0.5\sqrt{\Delta t}$ to $0.29\sqrt{\Delta t}$ (a 42.40% decrease for a fixed signal rate of 0.5 photons). Notably, when the background photon rate is negligible (*λ*_bg_ ≪ *λ*_I_), the SNR also exhibits a square-root dependence on signal intensity. These numerical simulation results agree with previous work.^41^^,^^42^

In the spectral domain, the SSNR displays a linear dependence on exposure time. This is displayed in Figure 2, which depicts a static line object with dimensions of 6 μm in height and 0.12 μm in width. Figure 2a presents a horizontal line imaged at increasing exposure durations, and the corresponding SSNR maps computed using Equation S42 are displayed in Figure 2b. A progressive increase in SSNR is observed with longer exposures, as reflected by the brighter intensities across the maps. The radially averaged SSNR as a function of radial frequency, ⟨SSNR⟩(*ρ*), is shown together with the estimated background curve SSNR_bg_ and the cutoff frequency *ρ*_c_, which is identified by the cross in Figure 2c. For the exposure times of 10, 50, and 100 ms, we observe the cutoff frequencies increase from 3.86 to 4.31 and 4.47 μm^−1^ (averaged over angles using the values from Table S2), respectively, which signifies the increase in the image resolution by 11.65% and 15.80% due to the signal accumulation. Moreover, the total SSNR measured by the area under the curve AUC(0,*ρ*_c_) rises from 1,400 to 7,000 and 14,000 (averaged over angles) for the respective exposures, demonstrating a 400% (5-fold) and 900% (10-fold) increase in the total SSNR accumulated in the image.

**Figure 2:**
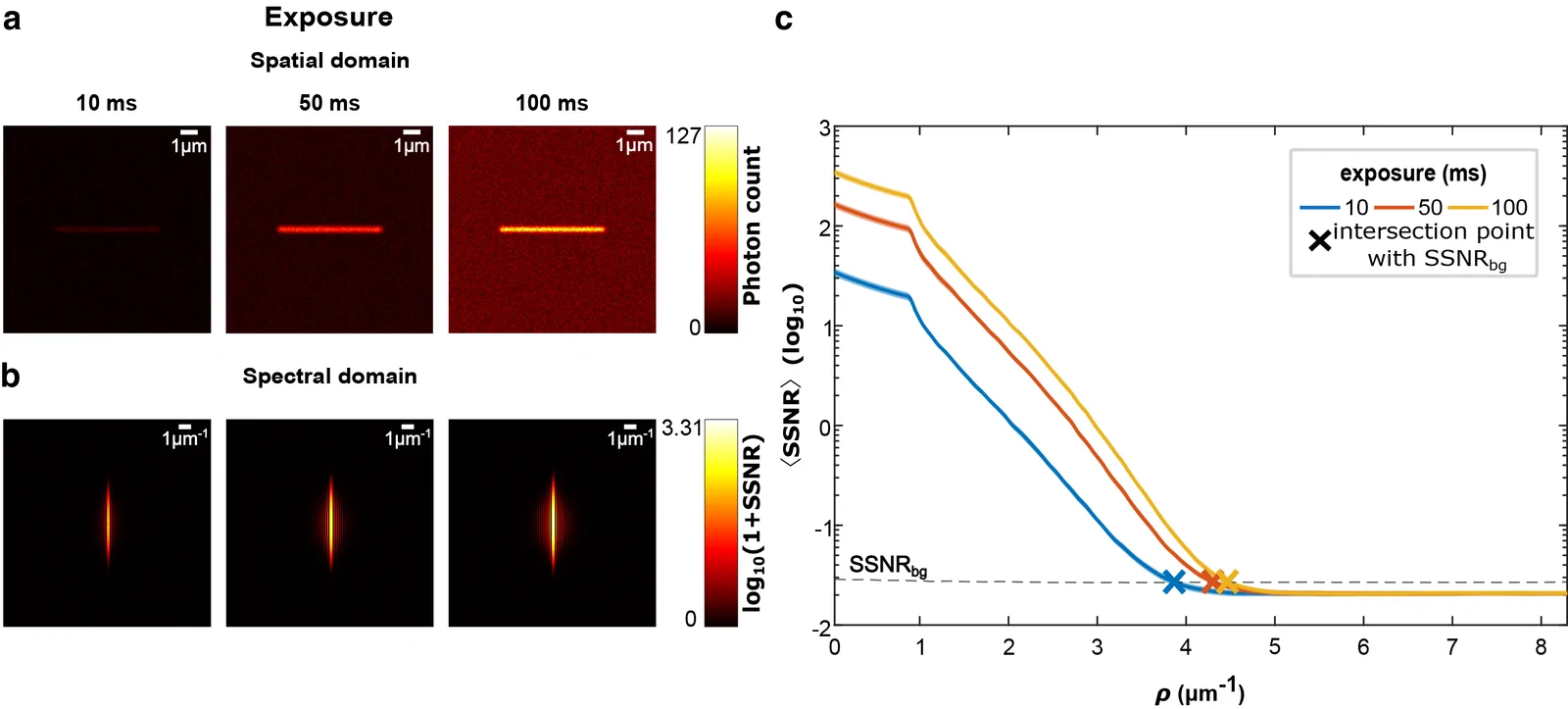
Increased exposure time improves SSNR for static objects. (a) Simulated images of a line object of height 6 μm and a width of 0.12 μm, oriented horizontally at exposure times of 10, 50, and 100 ms. As the signal accumulates over time, the object appears brighter relative to the background. (b) The corresponding SSNR maps reveal that the spectral distribution aligns orthogonally to the line orientation. Due to linear dependence of SSNR on exposure time, the horizontal spectral component becomes increasingly dominant when the data are normalized and displayed on a logarithmic scale. (c) Radially averaged SSNR curves are shown for exposure times of 10, 50, and 100 ms. The frequency cutoffs, marked by cross symbols at the intersections of the SSNR curves with the background SSNR level (dashed black line), shift toward higher frequencies as exposure time increases. At the same time, the radial average curve increases across the whole bandwidth with the increase in exposure time.

To ensure consistency between computational and theoretical findings, we compare our numerical results to the derivations presented in Note S2. When both velocity components *v*_*x*_ and *v*_*y*_ in Equation S15 are zero, there is no sinc modulation affecting the signal during static acquisition, and the SSNR increases linearly with exposure duration for all spatial frequency bands. Specifically, the peak magnitudes of SSNRs at an exposure time of 100 ms increase by 0.30 and 1.00 on a logarithmic scale compared to 50 and 10 ms, respectively. These results correspond to the linear scaling factors of 2 and 10 for exposure times of 50 and 10 ms, respectively. Our numerical experiments verify this theoretical prediction, showing that as exposure time increases, the SSNR consistently rises and eventually surpasses the average background SSNR at higher frequencies, thus improving estimated resolution.

### Effect of translational motion on line object imaging

We simulated the line object of 6 μm in height and 0.12 μm in width from Figure 2 under various angular inclinations (0°, 45°, and 90° in the clockwise direction). For each line of different orientation, we applied translational motion across the *x* axis to the object at a constant velocity *v*_*x*_ = 40 nm/ms, which matches the velocities observed in biological processes listed in Note S3.6.

Figure 3a displays the accumulated images of the line for exposure times of 10 and 100 ms. As the exposure time increases, the line elongates horizontally along the direction of motion. For the 0° inclination, the line becomes thicker (6 × 4 μm), gradually approaching a square shape. At a 45° inclination, the long exposure forms a parallelogram shape with a width of 4 μm. For the 90° angle, the line width extended from 6 to 10 μm while the line height remains preserved. The resulting elongation occurs due to the accumulation of object snapshots during translation along the motion direction. As a result, the motion of the 90° line results in a significant overlap of the signal photon accumulations over time, causing the visible extension to disappear. For the same reason, the 90° line reached a maximum photon count of 125 photons per pixel, compared to 46 and 47 photons per pixel for the 0° and 45° lines, respectively.

**Figure 3:**
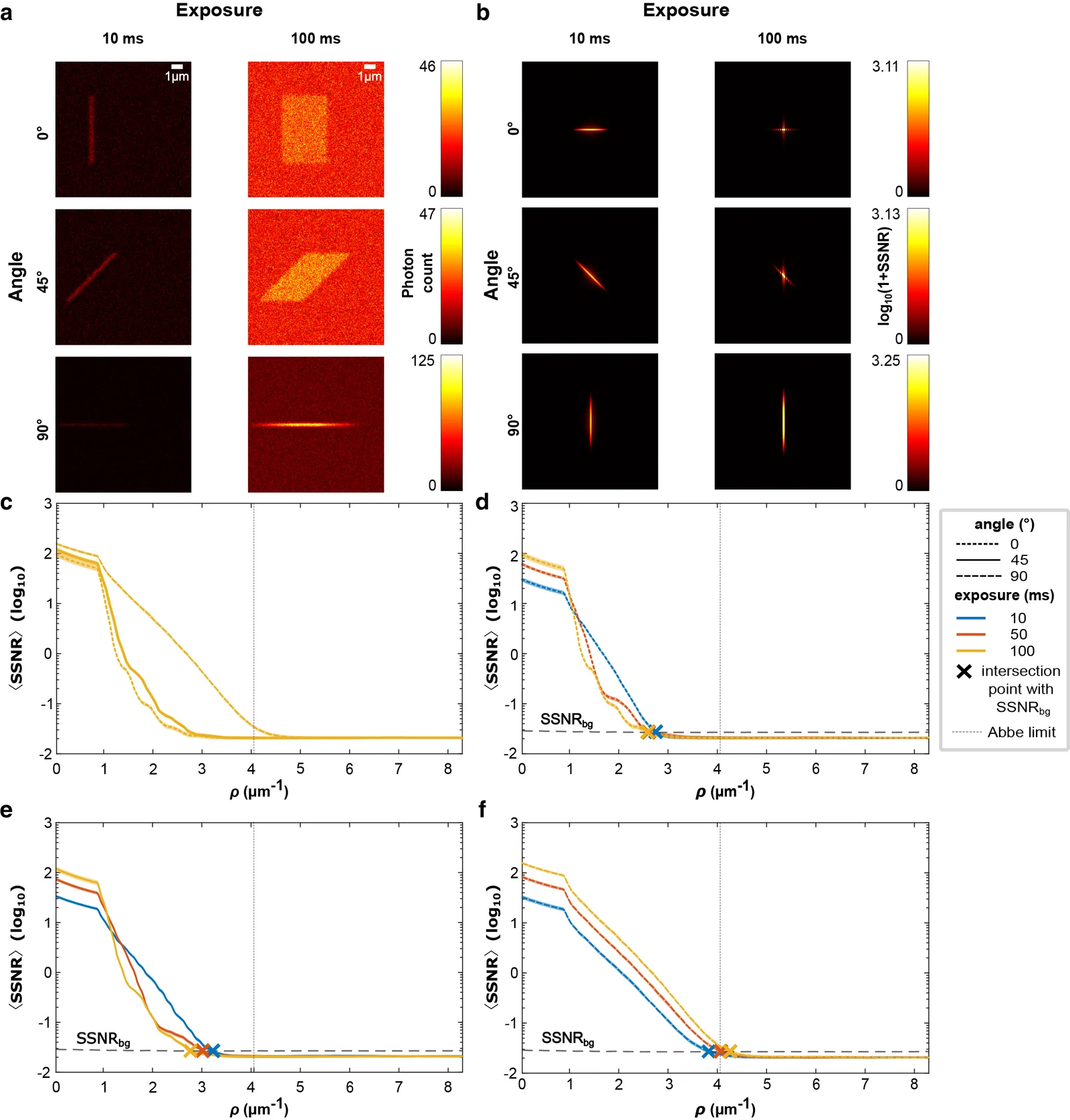
Translational motion blur on line objects with various inclinations. (a) Measured images of a line object oriented at 0°, 45°, and 90° under horizontal translation during acquisition. Two exposure times are shown: short (10 ms) and long (100 ms). The 0° and 45° lines exhibit a larger horizontal elongation after the long exposure time, while the 90° line preserves most of its structural integrity. (b) Corresponding SSNR maps indicate that horizontal motion broadens the spectrum along the vertical motion axis, most prominently for the 0° and 45° lines, whereas the spectrum for the 90° line remains relatively unchanged. (c) Radial averages of SSNR versus spatial radial frequency for long exposure time (100 ms). The semi-transparent bands indicate the interval spanning one standard deviation below and above the average SSNR. The 90° line demonstrates a higher radial average SSNR than that of the 0° and 45° lines. (d–f) Radial SSNR averages at varying exposures (10, 50, and 100 ms) for 0° (d), 45° (e), and 90° (f) angles. The 90° line, collinear to the motion, shows the greatest improvement in SSNR radial averages over increased exposures; the 0° and 45° lines show the redistribution of SSNR in the radial frequency spectrum with the increase in exposure time. Additionally, the gray vertical line depicts the Abbe diffraction limit, which remains fixed with the change in exposure time.

In the frequency domain, the SSNR pixel maps (Figure 3b) illustrate the spectral modifications induced by motion. At an exposure time of 10 ms, the 0° line exhibits a spectrum concentrated along the *f*_*x*_ direction. When the exposure increases to 100 ms, a vertical frequency component emerges, indicating spatial blurring along the motion direction.

This effect stems from sinc modulation (see Equation S15), which suppresses frequencies aligned with motion and relatively emphasizes those in the orthogonal direction throughout the entire spectrum. A similar trend is observed for the 45° line: a single frequency component at 10 ms evolves into a dual-component structure at 100 ms. In contrast, the 90° line at 100 ms shows a constructive spectral effect: horizontal components diminish, and the intrinsic vertical spectrum of the object becomes more pronounced. This behavior results from the alignment between the object’s dominant spectral axis and the motion direction, as described by the sinc-shaped modulation function. Since only the *f*_*x*_ component is modulated (*v*_*y*_ = 0; see Equation S15), the intrinsic spectrum of the line remains largely intact. The main spectral lobe, which contains 82.55% of the total SSNR and occupies 25% of the spectral domain vertically and 5% horizontally, is minimally influenced by this modulation.

As we examine the ⟨SSNR⟩ curves at a fixed exposure time of 100 ms, shown in Figure 3c, we find that the 90° line contains an AUC of 6,200, compared to 3,900 and 3,100 for the 0° and 45° cases, respectively. This constitutes 37% and 50% loss of the SSNR due to the respective orientation change. Similarly, the cutoff frequencies *ρ*_*c*_ = 2.58 μm^−1^ for the 0° line and 2.77 μm^−1^ for the 45° line are 39% and 35% lower compared to the 90° line cutoff at 4.26 μm^−1^, respectively. These results emphasize that object orientation with regard to the translational motion direction can significantly degrade the SNR by up to 50% and the image resolution by up to 40%.

Furthermore, as Figure 3f displays, the ⟨SSNR⟩ curve of the 90° line is *elevated* across the whole spectrum, with both *ρ*_*c*_ and AUC(0,*ρ*_c_) increasing as exposure times increase from 10 to 50 and 100 ms. Specifically, the frequency cutoffs rise from 3.84 to 4.08 and 4.26 μm^−1^ (+11%), and the AUC(0,*ρ*_c_) increases from 1,300 to 3,300 and 6,200 (+370%). Compared to the ⟨SSNR⟩ values obtained for a static line (Figure 2), the increase in frequency cutoffs under motion is lower than that of the static line (11% against 16%). Analogous results can be observed for the total SSNR, with an increase of 370% against 900%. These results highlight that motion blur degrades resolution even when the motion is predominantly aligned with the object orientation.

However, for 0° and 45° lines, increasing the exposure time *redistributes* the average radial frequency content contained in the ⟨SSNR⟩(*ρ*), as shown in Figures 3d and 3e. Upon visual inspection, we observe the presence of an “inflection frequency” *ρ*_infl_, wherein the ⟨SSNR⟩(*ρ*) curves for various exposures intersect. We calculate the inflection frequencies as the minimum variance point between the ⟨SSNR⟩(*ρ*) curves and arrive at *ρ*_infl_ values of 0.951 and 1.551 μm^−1^ for 0° and 45° lines. We then compare the total SSNR for frequencies below the inflection point with AUC(0,*ρ*_infl_) and above it with AUC(*ρ*_infl_,*ρ*_c_). With exposure times growing from 10 to 100 ms, we observe a consistent increase of 210% in AUC(0,*ρ*_infl_) for both 0° and 45° lines. In contrast, for frequencies above the inflection point, the AUC(*ρ*_infl_,*ρ*_c_) *decreases* by 28% and 79% for the same angles.

Moreover, we can observe from Figures 3d–3f that the frequency cutoffs *ρ*_c_ increase with exposure time for the 90° line, ranging from 3.84 to 4.26 μm^−1^ (+10.94%) for the 10 and 100 ms exposures, respectively. For the 0° line, it falls from 2.76 to 2.58 μm^−1^ (−6.52%), and for the 45° line, it decreases from 3.22 to 2.77 μm^−1^ (−13.98%). This orientation-dependent pattern repeats the high-frequency total SSNR redistribution pattern, highlighting the resolution dependence on orientation, a property intrinsic to the object.

To make a direct comparison between the static and dynamic cases across all angles and exposure times, we collected the AUC(0,*ρ*_c_) and *ρ*_c_ frequency metrics for both objects side by side in Table S8. These values correspond to the radially averaged SSNR curves shown in Figure 2c for the fixed line and in Figures 3d–3f for the translating line, allowing a direct visual and numerical comparison between the two cases. Consistent with the trends discussed above, the fixed line’s AUC(0,*ρ*_c_) and *ρ*_c_ exceed those of the translating line at every angle and exposure time, with the gap narrowing substantially at 90°: at 100 ms, the translating line’s AUC is 55% lower and its *ρ*_*c*_ is 4.7% lower than the fixed line, compared to losses of 77% and 42% at 0° and 73% and 38% at 45°, respectively. The relative growth of AUC(0,*ρ*_*c*_) with exposure time is markedly steeper for the fixed line than for the translating line at all angles, exhibiting the effect of motion blur: from 10 to 100 ms, the fixed line’s AUC increases by 890%, 910%, and 890% for the 0°, 45°, and 90° lines, respectively, whereas the translating line’s AUC increases by only 180%, 200%, and 370% over the same interval. This corresponds to the fixed line’s relative AUC growth being roughly two to five times larger than that of the translating line, with the disparity most pronounced at 0° and least pronounced at 90°, where the object orientation is aligned with the direction of motion. The cutoff frequency *ρ*_*c*_ increases modestly and monotonically with exposure for the fixed line, rising by 16% at all three angles from 10 to 100 ms, but only does so for the translating line at 90°, where it increases by 11% over the same interval, decreasing instead at 0° (−6.5%) and 45° (−14%), in line with the orientation-dependent redistribution described above.

Additionally, we benchmarked SSNR-based resolution estimates with two-image and single-image FRC to verify the consistency of the results with other existing techniques. This comparison is presented in Tables S3 and S4.

### Effect of Brownian motion on point objects

Figure 4a presents the simulated measurements of diffusing point objects acquired at exposure times of 10 and 100 ms for diffusion coefficients of 3 and 6 μm^2^/s. Upon visual inspection, increasing the diffusion coefficient from 3 to 6 μm^2^/s results in a broader spatial distribution of the detected photons and a reduction in the peak intensity. Specifically, the maximum signal intensity decreases by 47.22%, indicating a reduced photon accumulation within individual pixels due to the increased stochastic displacement of the emitter during the exposure period.

**Figure 4:**
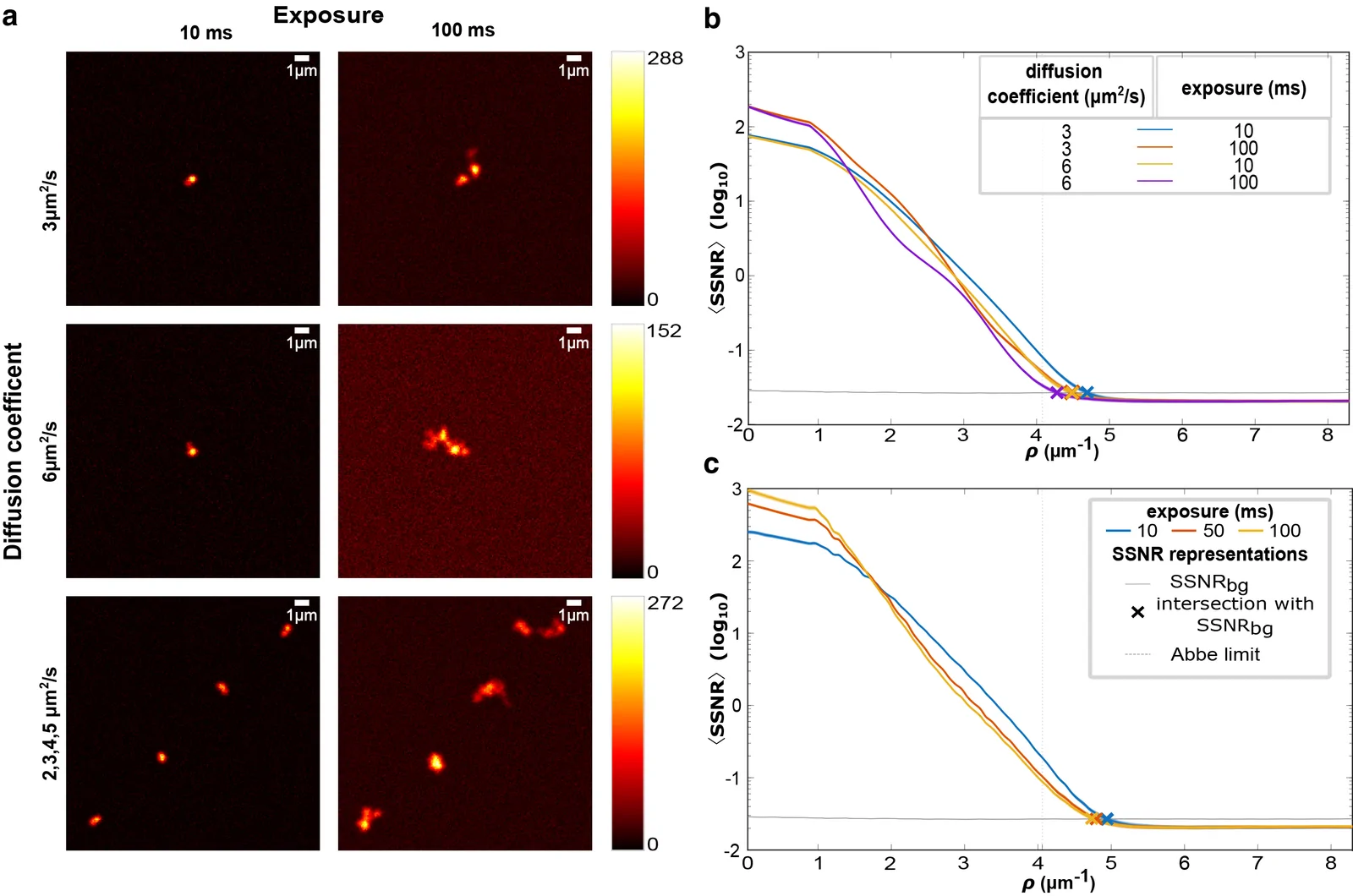
Brownian motion on point objects with varying diffusion coefficients and exposure times. (a) Measured images of point objects undergoing Brownian motion. The top two rows show a single object with diffusion coefficients (D) of 3 and 6 μm^2^/s, respectively, each imaged at 10 and 100 ms exposure. The bottom row shows multiple objects with diffusion coefficients of 2, 3, 4, and 5 μm^2^/s at the same two exposures. For all objects, the 100 ms exposure produces markedly more pronounced motion blur than the 10 ms exposure. For the lower diffusion coefficient, the 100 ms measurement also shows higher background contrast compared to the higher diffusion coefficient, consistent with the higher photon accumulation indicated by the corresponding colorbar maxima. (b) ⟨SSNR⟩ profiles for the two single-point objects in (a) (D = 3 and 6 μm^2^/s), each shown at 10 and 100 ms exposure. The cutoff frequency is lowest for the combination of the highest diffusion coefficient and longer exposure (purple curve). An inflection point is observed in both cases: between the blue and red curves (D = 3) and between the purple and yellow curves (D = 6). Doubling the diffusion coefficient, along with a 10-fold increase in exposure time (red versus yellow), yields comparable cutoff frequencies. (c) ⟨SSNR⟩ profiles for the multiple-point object shown in the bottom row of (a), at exposures of 10, 50, and 100 ms. As with (b), the cutoff frequency is defined by the intersection with SSNR_bg_, marked by ×.

In the frequency domain, increasing the exposure time leads to a progressive degradation of the image resolution for both diffusion coefficients. For the object diffusing at 3 μm^2^/s, extending the exposure time from 10 to 100 ms decreases the cutoff frequency *ρ*_c_ from 4.69 to 4.49 μm^−1^, corresponding to a reduction of 4.4%. A similar trend is observed for the diffusion coefficient of 6 μm^2^/s, where *ρ*_c_ decreases from 4.49 to 4.28 μm^−1^, representing a loss of 4.7%. These results demonstrate that both longer exposure times and stronger diffusion contribute to the loss of resolvable spatial frequencies. Notably, the cutoff frequency obtained for the 3 μm^2^/s object at 100 ms exposure (4.49 μm^−1^) is identical to that measured for the 6 μm^2^/s object at 10 ms exposure, indicating that the effects of increased diffusion and prolonged exposure produce a comparable degradation of the attainable resolution.

For the multiple-point object shown in the bottom row of Figure 4a, an analogous behavior can be observed. Increasing the exposure time from 10 to 100 ms decreases the cutoff frequency *ρ*_c_ from 4.94 to 4.74 μm^−1^, corresponding to a reduction of 4.1%. However, examination of the ⟨SSNR⟩(*ρ*) curves reveals that the reduction in cutoff frequency is accompanied by a redistribution of SSNR across the spectrum. To quantify this behavior, we calculate the AUC below the inflection frequency, AUC(0,*ρ*_infl_), and above it, AUC(*ρ*_infl_,*ρ*_c_). We observe that AUC(0,*ρ*_infl_) increases from 8.77 × 10^3^ to 22.85 × 10^3^ and subsequently to 3.50 × 10^4^ for exposure times of 10, 50, and 100 ms, respectively. This corresponds to increases of 160% between 10 and 50 ms and a further 54% between 50 and 100 ms. In contrast, AUC (*ρ*_infl_,*ρ*_c_) decreases from 1.21 × 10^3^ to 9.50 × 10^2^ and further to 8.3 × 10^2^ for exposure times of 10, 50, and 100 ms, respectively, corresponding to reductions of 21% between 10 and 50 ms and an additional 13% between 50 and 100 ms. Although the decrease in cutoff frequency from 4.94 to 4.74 μm^−1^ is modest (4.1%), the corresponding uncertainty intervals, [4.87,5.04] and [4.69,4.82] μm^−1^, do not overlap, indicating that the reduction is statistically significant. However, the change in *ρ*_c_ remains small compared with the much larger changes observed in the AUC metrics. More importantly, the 4-fold increase in AUC(0,*ρ*_infl_), from 8.77 × 10^3^ to 3.50 × 10^4^, together with the 1.5-fold decrease in AUC(*ρ*_infl_,*ρ*_c_) from 1.21 × 10^3^ to 8.3 × 10^2^, demonstrates that longer exposure times primarily redistribute SSNR from higher to lower spatial frequencies. Thus, the dominant effect of Brownian motion in this regime is the redistribution of spectral information rather than a major decrease in *ρ*_c_.

### Effect of rotational motion on Siemens star object imaging

To investigate the influence of rotational motion on image resolution based on SSNR radial averages, we simulated a Siemens star pattern with three spokes and applied clockwise rotational motion at angular velocities of 10.48, 6.98, and 5.24 radians/s, which correspond to rotation periods of 600, 900, and 1,200 ms, respectively. These periods correspond to increasing angular displacements of 60°, 40°, and 30° for 100 ms of exposure time. Figure 5a displays the accumulated images of the rotating Siemens star for exposure times of 10, 50, and 100 ms. With increased exposure time, the spokes become increasingly blurry, particularly at larger radial distances from the center of rotation, an effect commonly referred to as angular blurring or rotational motion blur.^43^ This effect is further amplified by the higher angular velocities for a fixed exposure time.

**Figure 5:**
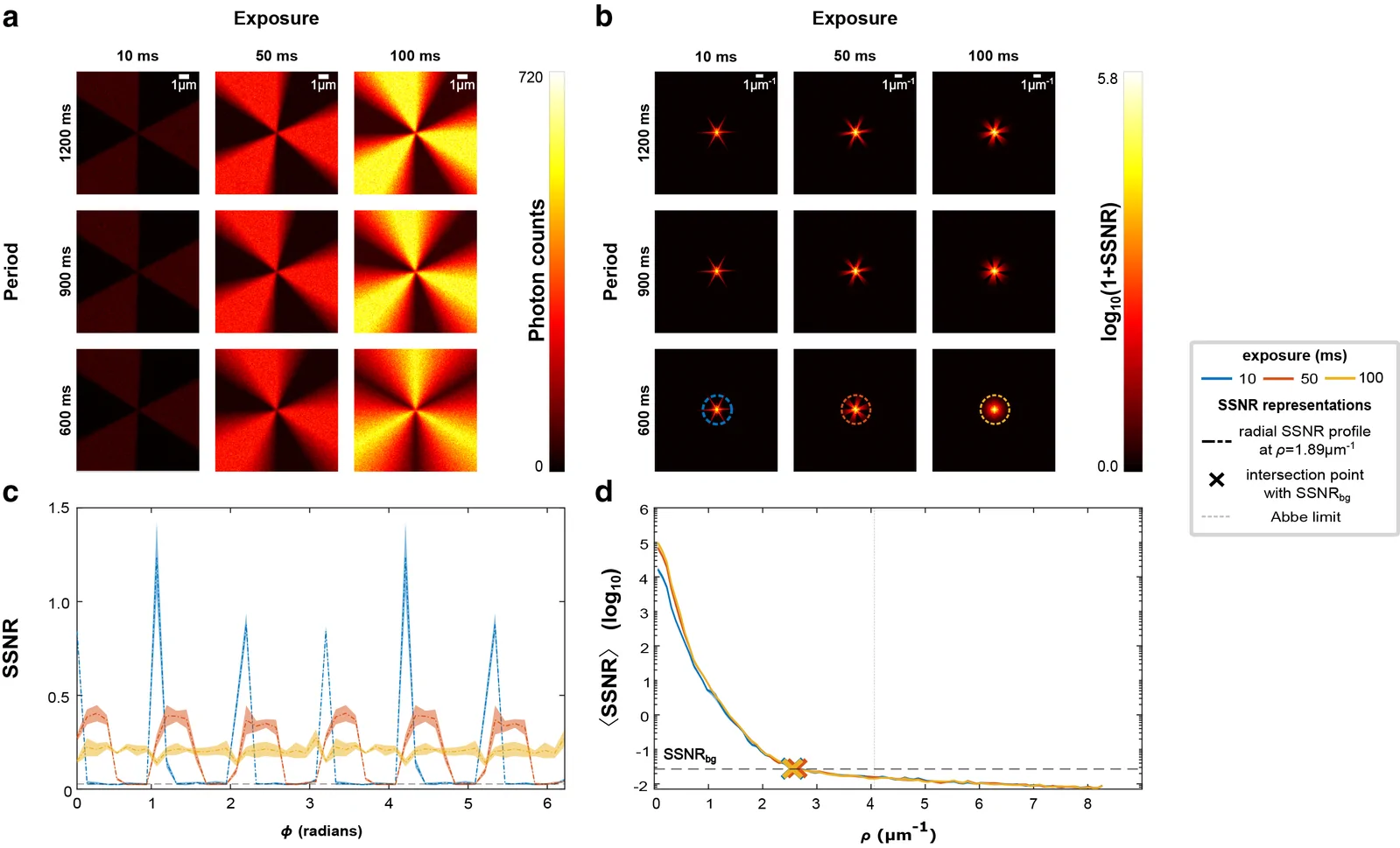
Rotational motion blur effects are concealed in SSNR radial averages yet visible in radial profiles. (a) Reconstructed images of a Siemens star with three spokes rotating during acquisition with exposure times of 10, 50, and 100 ms. The angular blur of the spokes increases with both exposure duration and angular velocity (the inverse of the rotation period), due to the greater accumulated angular displacement during exposure. This effect scales with radial distance, producing more pronounced blurring at larger radii. (b) SSNR maps expose the angular dependence of this blur: SSNR in high-frequency regions, originally confined to narrow spokes, broadens in the angular direction with increasing exposure and angular velocity. (c) Radial slices (indicated by dashed circles in b) at *ρ* = 1.89 μm^−1^ directly capture the angular blurring effect, with peaks broadening markedly as the exposure time increases. The dashed line in (c) shows the mean across multiple realizations, while the corresponding semi-transparent band indicates the interval spanning one standard deviation above and below the mean. (d) In contrast, full radial averages conceal the angular blur: all curves rise at low frequencies and converge to a similar cutoff, thereby masking the angular degradation evident in (a)–(c). For a stationary object, longer exposures would also increase the high-frequency SSNR; however, rotational motion suppresses this gain by redistributing SSNR across angles. The curves are logarithmically scaled. We provide linearly scaled plots in Figure S1 to further highlight the differences between different exposure times.

The frequency-domain representation displays a similar pattern. The SSNR pixel maps in Figure 5b display six narrow angular frequency components around the center for an exposure time of 10 ms. With an increase in exposure time and angular velocity, these components create motion blur in the clockwise direction. This demonstrates the spatial frequency redistribution effect caused by rotational motion.

To quantify this effect, we investigated the SSNR radial profile at a fixed radial frequency *ρ* = 1.89 μm^−1^, SSNR(*ρ* = 1.89 μm^−1^, *ϕ*), and a period of 600 ms (Figure 5c). At an exposure time of 10 ms, the profile rises to SSNR values of 1.25, followed by a rapid drop to a near-background SSNR value of 0.027 after an average nonbackground angular spread of 0.1676 radians, which constitutes 16.76% of the angular range. At 50 ms, the peak SSNR value decreases to around 0.35, but the angular spread broadens to about 0.5445 radians on average (52% of the angular range). For the 100 ms exposure time, the SSNR radial profile remains flattened across the entire 6.2832 radian range, centered at an SSNR value of 0.027, with no notable drops. Overall, we observe an increase in angular blurring with exposure time.

The radial average ⟨SSNR⟩ in Figure 5d shows an increase in AUC(0,*ρ*_c_) from 3.4 × 10^4^ to 1.4 × 10^5^ and from 18.99 × 10^4^ to 1.9 × 10^5^ for exposures of 10, 50, and 100 ms, accounting for 300% and 460% increases in total SSNR. As the curves shown in Figure 5d appear visually similar due to logarithmic scaling, we added a linearly scaled plot in Figure S1 to elucidate the exposure time effects. The difference between the curves is predominantly pronounced for spatial frequencies below 1 μm^−1^, resulting in a 460% increase in AUC for exposures from 10 to 100 ms. For the frequencies above 1 μm^−1^, the total SSNR increases by just 12%, and no clear ordering of the cutoff frequencies can be observed, as they yield values around *ρ*_c_ = 2.6 μm^−1^ (±0.05 μm^−1^). Therefore, it can be concluded that the rotational motion blur-induced SSNR redistribution is mostly concentrated in lower frequencies and thus cannot be detected by the cutoff frequencies alone.

Additionally, we explore how rotational velocity influences resolution changes. For an exposure time of 100 ms, we observed the angular spread of the radial profile SSNR(*ρ* = 1.89 μm^−1^, *ϕ*) increase from 0.59 for the period of 1,200 ms to 0.71 for the period of 900 ms, constituting a growth of 0.12 radians. Furthermore, for an exposure time of 50 ms, the angular spread growth is smaller, at 0.08 (from 0.34 to 0.42 radians), for the same rotational periods. No measurable increase in angular blur was apparent at an exposure time of 10 ms (0.17 radians across all periods), suggesting that such brief exposure is sufficient to preserve the structural integrity of the object. Further details of this effect, shown across all exposure times and periods, are presented in Table S7.

### Effect of motion on fluorescence microscopy data

To assess whether the spectral redistribution observed in the simulations is also present in experimental measurements, we analyzed dynamic light-sheet microscopy data of the *Drosophila* endoderm during embryogenesis acquired by Keenan et al.^44^ A lateral slice at a fixed axial position was selected to investigate the influence of motion blur on the measured image spectrum. To demonstrate the effect of extended integration times, consecutive image frames were summed with factors of 5 and 10, resulting in frames with effective exposure times of 55, 275, and 550 ms. The original image data used in this analysis are shown in Figure S3.

The corresponding measurements are presented in Figure 6a. As the effective exposure time increases, the maximum image intensity rises from 7,562 to 19,676 and subsequently to 23,892 counts. However, this increase does not follow the expected proportional relationship from exposure-time scaling. Specifically, while the exposure time increases by factors of 5 and 10, the maximum intensity increases by only 160.19% and 215.96% relative to the 55 ms acquisition. This behavior indicates that the signal accumulation process is only partially constructive due to the ongoing motion of the biological structures during image formation, causing photon contributions from different time points to spread across neighboring spatial locations. The corresponding radially averaged ⟨SSNR⟩(*ρ*) curves were then presented in Figure 6b and compared across exposure conditions. Upon visual inspection of the ⟨SSNR⟩(*ρ*) curves, we observed the presence of an inflection frequency *ρ*_infl_, defined as the minimum variance point between the curves obtained at different exposure times. Due to the absence of background in the light-sheet microscopy data, we defined *ρ*_c_ as the upper frequency limit corresponding to the largest radial bin center, i.e., the Nyquist frequency at *ρ*_c_ = 4.9609 × 10^−4^, since the absence of a measurable background in the light-sheet microscopy data precluded the determination of a cutoff frequency based on the SSNR threshold criterion. To quantify the redistribution of SSNR, we calculate the AUC below the inflection frequency, AUC(0,*ρ*_infl_), and between the inflection and upper frequency limit, AUC(*ρ*_infl_,*ρ*_c_). Using *ρ*_infl_ = 5.08 × 10^−5^, we find that AUC(0,*ρ*_infl_) increases from 4.39 × 10^11^ to 1.45 × 10^12^ and subsequently to 1.88 × 10^12^ for exposure times of 55, 275, and 550 ms, respectively. This corresponds to an increase of 230.30% between 55 and 275 ms, followed by a further increase of 29.66% between 275 and 550 ms. In contrast, the high-frequency contribution represented by AUC(*ρ*_infl_,*ρ*_c_) decreases from 2.81 × 10^9^ to 1.87 × 10^9^ and 1.54 × 10^9^ over the same exposure range. These values correspond to reductions of 33.45% and 17.65%, respectively. Therefore, while increasing the effective exposure time enhances the accumulation of low-frequency SSNR, it simultaneously reduces the contribution of higher-frequency components. This behavior closely resembles the spectral redistribution observed for simulated moving objects, indicating that motion blur during the imaging of dynamic biological processes progressively shifts the available SSNR toward lower spatial frequencies.

**Figure 6:**
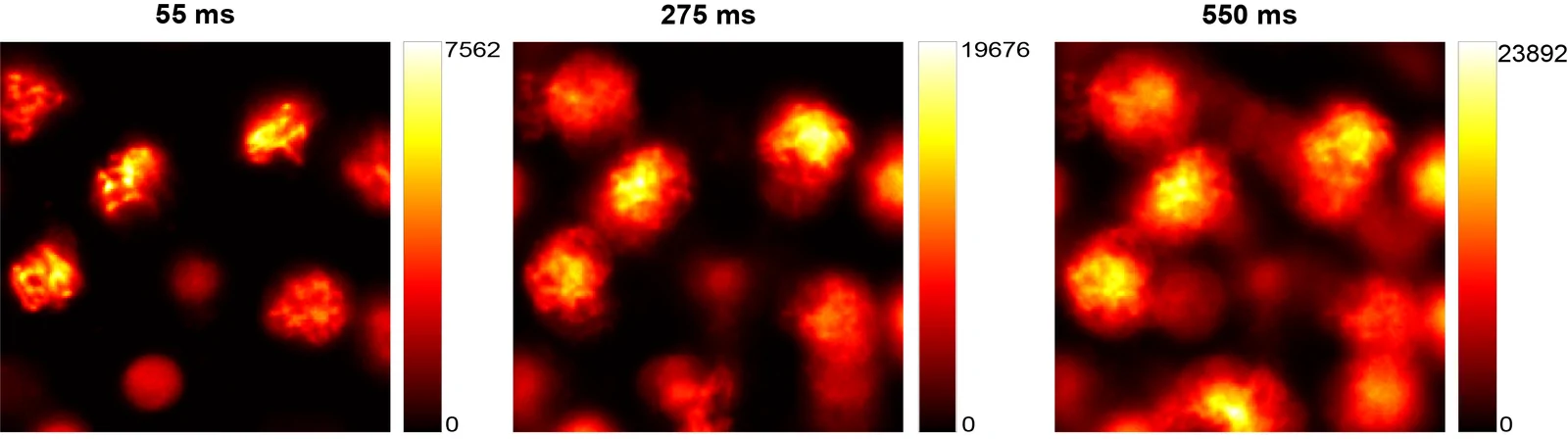
SSNR analysis of a dynamic *Drosophila* light-sheet fluorescence microscopy dataset. (a) Measurements at increasing cumulative exposure times (55, 275, and 550 ms), with photon accumulation reflected in the corresponding colorbar maxima. Neighboring structures, well separated at 55 ms, increasingly merge at longer cumulative exposures due to motion blur. (b) Redistribution of SSNR within the spectrum shown via radial SSNR averages, ⟨SSNR⟩, for the same cumulative exposure times. An inflection frequency is observed around *ρ* = 0.04 μm^−1^, beyond which the ordering of SSNR content across exposure times reverses. Near *ρ* = 0.3 μm^−1^, the curves begin to merge, exhibiting near-identical behavior at higher frequencies. For reference, the Abbe diffraction limits of the detection optics (NA = 1.0, *λ* = 488 nm) correspond to spatial frequencies of approximately 4.1 μm^−1^, far beyond both the Nyquist frequency imposed by the 1 μm sampling and the spectral range shown. Consequently, the plotted SSNR behavior is constrained by sampling and motion blur rather than by the optical cutoff.

## Conclusion

In this study, we have demonstrated that resolution is a dynamic variable governed by the fundamental trade-off between signal accumulation and object motion. By developing a simulation framework coupled with the SSNR metric, we quantified how these dynamics alter the information content of an image.

We systematically investigated how motion blur affects spatial information by simulating time-dependent object measurements and evaluating total SSNR and resolution cutoffs under dynamic conditions. First, we established a baseline by measuring SSNR for the static line object, where an exposure increase from 10 to 100 ms showed a 10-fold improvement in total SSNR and a resolution increase of 15.80%. However, under translational motion along the +*x* direction, lines oriented at 0° and 45° relative to the motion direction exhibit SSNR redistribution around an inflection frequency *ρ*_infl_: the SSNR is boosted by over 200% for frequencies below *ρ*_infl_ and eroded by up to 80% for frequencies above *ρ*_infl_.

Furthermore, we observe that object orientation influences the changes in resolution that occur with increasing exposure time. Increasing the exposure time from 10 to 100 ms, the cutoff frequency of the 90° line increases by 10.94%, but for the 0° and 45° lines, it falls by 6.52% and 13.98%. This effect reflects the spectral orientation dependence: 0° and 45° lines concentrate SSNR along the horizontal frequency direction *f*_*x*_ and thus suffer from motion-induced sinc modulation, whereas the 90° line, whose spectrum is localized in the vertical frequency direction *f*_*y*_, remains largely unaffected. Notably, the increase in cutoff frequency observed for the 90° line represents an unusual scenario, in which motion has a constructive rather than degrading effect. We propose that this “constructive drift” phenomenon could be exploited as a novel, deliberate acquisition strategy in future research. For example, for highly anisotropic structures whose dominant orientation is known, inducing a controlled stage drift parallel to that orientation could, in principle, act as a physical low-pass filter, smearing isotropic background noise while actively accumulating constructive signal for the targeted structures. The practical realization of such an approach would require prior knowledge of both the structural orientation and the imposed and inherent motion trajectory. In typical imaging scenarios, however, object spectra are more isotropic rather than concentrated along a single axis, and the underlying biological motion is generally unknown rather than externally controlled. In such cases, motion attenuates high-frequency components, and the fine spatial details they encode are irretrievably lost due to motion blur. Without reliable structural or motion priors, a motion-blurred observation is indistinguishable from an image of a static object exhibiting the same apparent morphology, creating an inherent ambiguity in the interpretation of biological structures.

Additionally, our results highlight that the resolution metrics are strongly dependent on the intrinsic properties of the imaged object. We showed that varying line orientation from 90° to 0° for a fixed translational velocity of 40 nm/ms in the +*x* direction at a fixed exposure of 100 ms resulted in degradation of the total SSNR by up to 50% and of the image resolution by up to 40%. Furthermore, we demonstrated that decreasing the rotational period from 1,200 to 900 ms results in angular blurring increases of 0.08 and 0.12 radians at fixed exposures of 50 and 100 ms, respectively, but does not produce any significant changes at an exposure of 10 ms. Additionally, our results demonstrate that stochastic motion can substantially affect both image resolution and the spectral distribution of information. We showed that increasing the diffusion coefficient from 3 to 6 μm^2^/s reduced the maximum signal intensity by 47%, while increasing the exposure time from 10 to 100 ms decreased the cutoff frequency by up to 4.7%. Furthermore, we found that longer exposure times progressively redistribute SSNR toward lower spatial frequencies, with low-frequency AUC increasing by up to 300% and high-frequency AUC decreasing by up to 31% over the investigated exposure range. Since intrinsic object properties such as velocity, rotational period, and diffusion coefficient cannot be directly controlled during real imaging scenarios, we recommend conducting exploratory imaging of representative samples to obtain reliable estimates of intrinsic parameters where possible. These estimates can be used to obtain a data-driven spatio-temporal prior, facilitating the detection of motion blur.

In addition, the analyses presented in this work were performed for both isolated objects and fields of view containing multiple objects. Provided that motion blur affects the entire field of view, the observed effects and the underlying mechanisms described here remain applicable. However, in situations where motion is spatially heterogeneous, such that only a subset of the field of view undergoes motion while other regions remain stationary, the proposed analysis may yield a relatively high global resolution estimate despite the presence of local motion blur. This behavior arises because the method is designed to identify the highest resolvable spatial frequencies present within the entire field of view, which may be dominated by stationary regions. Therefore, caution should be exercised when interpreting global resolution estimates in the presence of spatially nonuniform motion. In such cases, we recommend performing the analysis locally within multiple regions of interest to better characterize and detect motion-blur-induced resolution, similar to the procedure conducted in Zhao et al.^45^

Notably, the experimental dataset analyzed in this work represents an example of spatially heterogeneous motion, in which only a subset of image structures exhibited motion while the remainder of the field of view remained largely stationary. Nevertheless, we were still able to observe the characteristic redistribution of SSNR within a portion of the spatial-frequency spectrum because the contribution of the blurred structures was sufficiently strong relative to the static background. We do not suggest that such redistribution will necessarily be observed in all experimental datasets containing localized motion. Rather, our results indicate that when the motion-induced signal contribution is sufficiently pronounced, localized motion blur can manifest as a measurable redistribution in the SSNR profile, providing a means to detect its presence even in spatially heterogeneous scenarios. Furthermore, our results are broadly applicable to measuring motion-induced spectral attenuation, as they are supported by analytical derivations, numerical simulations encompassing multiple geometries and motion types commonly encountered in fluorescence microscopy, and experimental validation on biological data. While we demonstrated our methodology works on timescales often encountered in widefield microscopy, it is not inherently limited to these timescales. Given the generality of the simulation framework and the SSNR metric, we expect that our methodology can be generalized to super-resolved reconstructions as well. As seen in widefield microscopy, we expect SSNR maps to be equally promising for revealing motion blur in super-resolution microscopy.

Crucially, our results expose a significant blind spot in standard resolution metrics. We found that increased exposure times cause severe angular blurring in rotating structures, where the SSNR radial profiles showed an increase in blurring from 16% of the angular range to 52% and 100% as exposure increases from 10 to 50 and 100 ms. However, we also demonstrate that conventional radially averaged metrics, such as FRC, fail to detect this degradation because they mask angular motion blur. This creates a risk of having false confidence, where a researcher may believe they have achieved high resolution based on the metric, while the true underlying biological structure (e.g., a rotating bacterial flagellum or nodal cilia^46^) is severely degraded. Therefore, more work is required to develop resolution metrics for arbitrary motions, and we strongly recommend utilizing radial SSNR profiling rather than radial averages when analyzing rotationally dynamic specimens. Potential avenues for future research could include adapting existing metrics such as FRC^47^ and decorrelation analysis^48^ to measure the resolution of arbitrary geometries under arbitrary motion effects. Additionally, our analysis was limited to the effect of planar motion on lateral resolution. Extending the framework to investigate 3D motion and the associated effects of the 3D PSF remains an important direction for future work.

Furthermore, we note that our analysis was restricted to a shot-noise-dominated imaging regime and therefore assumes a Poisson noise model. This choice reflects the performance characteristics of modern sCMOS cameras, in which photon shot noise is frequently the principal noise source under typical fluorescence imaging experiments.^49^ Nevertheless, other imaging modalities or acquisition settings may be affected by additional noise sources, such as readout, dark-current, or detector-specific noise, which could alter the observed trade-offs and metric behavior.^50^ Extending the present framework to incorporate alternative noise models is an important direction for future work and will be necessary to assess the generality of our conclusions across different experimental configurations.

Minimizing movement-induced image degradations requires a methodological shift from solely maximizing SNR to optimizing the spatio-temporal trade-off between exposure duration and the motion blur in the sample. To achieve this, we suggest transitioning toward object-oriented, adaptive imaging paradigms. Recent advancements, such as the hybrid event-driven acquisition (hybrid-EDA) framework,^33^ have demonstrated the viability of using low-phototoxicity phase-contrast surveillance to monitor cellular dynamics in real time. By synthesizing our SSNR framework with such AI-driven multimodal hardware, future microscopes could continuously estimate the velocity vectors of a target structure using label-free surveillance. Upon detecting rapid movement, the system’s onboard AI would calculate the exact inflection frequency *ρ*_infl_ and instantly trigger high-intensity fluorescence lasers with an exposure duration strictly tuned to remain below this destructive spatio-temporal threshold.

This object-specific acquisition protocol balances SNR against motion blur, ensuring that photon accumulation remains entirely constructive. By establishing these absolute mathematical boundaries for kinematic degradation, our work provides a critical physical safeguard for structural fidelity, ensuring that super-resolved biological dynamics represent genuine optical measurements rather than hallucinated artifacts from computational over-reconstruction.

In practical terms, we recommend adopting an object-specific imaging approach based on our results to obtain accurate resolution estimates under dynamic movements. Object-specific properties, such as velocity estimates, can be obtained in an exploratory imaging step utilizing label-free phase-contrast surveillance to avoid unnecessary photobleaching. While doing so, we highlight the need to adjust resolution metrics to incorporate angular information rather than averaging it, thereby enabling more accurate resolution evaluation under rotational motion. We recommend employing a two-step acquisition protocol: an initial high-speed, low-SNR exploratory scan to estimate velocity vectors, followed by an acquisition where exposure time is tuned to the specific inflection frequency of the sample. Using this *a priori* knowledge with the adjusted resolution metrics will help determine an acquisition speed that balances SNR against motion blur. As a computational alternative to balancing SNR and exposure time, a promising direction could be to simultaneously image in separate slow, high-SNR and fast, low-SNR regimes, with subsequent computational extraction of relevant information. Such empirical exploration, utilizing dual-exposure priors, can guide the identification of optimal operating points specific to the imaging system without the need for an initial exploratory imaging step and could push the spatial and temporal boundaries of imaging fast biological processes.

## Data and code availability

The code that supports the findings of this study is openly available on GitHub^51^ at https://github.com/qnano/spatio-temporal-analysis. The light-sheet fluorescence microscopy data by Keenan et al.^44^ are publicly accessible as disclosed in the original publication (https://www.pnas.org/doi/full/10.1073/pnas.2112892119#data-availability).

## Acknowledgments

S.K., K.U., D.K., and C.S.S. were supported by the 10.13039/501100003246Netherlands Organisation for Scientific Research (NWO), under NWO Vidi project no. 20390.

## Author contributions

S.K., K.U., D.K., K.P., and C.S.S. designed the research. S.K. and K.U. derived the metric, implemented the experiments, analyzed the data, and wrote the initial draft manuscript. D.K. derived the simulation model. R.H. contributed discussion and ideas. The draft manuscript was edited by all authors. The study was supervised by C.S.S.

## Declaration of interests

The authors declare no competing interests.

## Supplemental information

Document S1. Figures S1–3, Tables S1–S8, Equations S1–S50, and supplemental materials and methods

Document S2. Article plus supplemental information
