# Supplementary material for: Quantifying the spatio-temporal image degradation under motion blur in fluorescence microscopy
Source: Biophys Rep (N Y). 2026 Sep 23;6(4):100290. doi: 10.1016/j.bpr.2026.100290 (PMC13677949; doi:10.1016/j.bpr.2026.100290)
Supplement: Supplementary file 1 [file mmc1.pdf]

Biophysical Reports, Volume *6*

Supplemental information

Quantifying the spatio-temporal image degradation under motion blur in fluorescence microscopy

Serafim Korovin, Kutay Ugurlu, Dylan Kalisvaart, Manon Kok, Rainer Heintzmann, Kirti Prakash, and Carlas S. Smith

1 The signal to noise ratio in fluorescence microscopy

In fluorescence microscopy, quantifying signal intensity relies on measuring the arrival rate of detected photons from a sample. While photons may be emitted at a constant *average* rate of λ photons per unit time, their detection follows an inherently stochastic process.

For an exposure duration Δt, the expected number of detected signal photons I in pixel i is given by:

$$\mu_{\mathrm{I},i} = \lambda_{\mathrm{I},i}\Delta t \tag{1}$$

However, due to statistical fluctuations, the actual number of detected photons in pixel i deviates from this expectation. These variations obey Poisson statistics, characterized by a standard deviation:

$$\sigma_{\mathrm{I},i} = \sqrt{\mu_{\mathrm{I},i}} = \sqrt{\lambda_{\mathrm{I},i}\Delta t} \tag{2}$$

This type of noise, also referred to as 'shot noise' or 'photon noise', arises from the fundamental randomness of photon detection. A critical implication is that, for an image based on the detection of μ_i photons, the theoretical per-pixel upper bound of the intensity-based signal-to-noise ratio (SNR) is:

$$\mathrm{SNR}_i = \frac{\mu_{\mathrm{I},i}}{\sigma_{i,I}} \tag{3}$$

A sufficiently high SNR is essential for resolving fine structural details in microscopy. The achievable SNR is fundamentally constrained by the photon statistics of the sample and the noise characteristics of the detector. This limitation is particularly significant in fluorescence imaging, where biological samples inherently impose a finite photon budget.

In practice, additional noise sources degrade the SNR beyond the theoretical shot noise limit. Multiple independent noise sources determine the total noise in an image, each contributing a standard deviation σ:

- **Background Photon Shot Noise**: In fluorescence microscopy, background signal contributions from autofluorescence, out-of-focus fluorescence, and stray light introduce additional noise. This background follows the same Poissonian behavior, with noise magnitude given by:

$$\sigma_{\mathrm{bg},i} = \sqrt{\lambda_{\mathrm{bg},i}\Delta t} \tag{4}$$

- **Dark Noise**: Dark noise arises from thermally generated charge carriers within the detector. This noise increases with exposure duration and follows Poisson statistics:

$$\sigma_{\mathrm{dark}} = \sqrt{D_i\Delta t} \tag{5}$$

  where D represents the dark current (thermal charge generation rate), and Δt is the exposure time.

- **Readout Noise**: Readout noise originates from the electronics responsible for digitizing the signal. Unlike the aforementioned noise sources, it does not scale with exposure time and is typically approximated as a constant ($\sigma_{\mathrm{read}} = \mathrm{constant}$.)

In state-of-the-art scientific CMOS (sCMOS) cameras, the impact of dark noise and readout noise is significantly reduced compared to traditional CCD sensors. Modern sCMOS detectors exhibit exceptionally low dark current due to advanced sensor cooling and fabrication techniques, rendering dark noise negligible under typical imaging conditions. Similarly, readout noise in contemporary sCMOS cameras is minimized through optimized readout circuitry and high-speed digitization, ensuring that its contribution remains insignificant relative to photon shot noise. This simplifies the total noise in an image pixel as the sum of the photon shot noise and background fluorescence:

$$\mathrm{SNR}_i = \frac{\lambda_{\mathrm{I},i}\Delta t}{\sqrt{\lambda_{\mathrm{bg},i}\Delta t + \lambda_{\mathrm{I},i}\Delta t}} \tag{6}$$

2 Spectral Analysis of Translational and Rotational Motion Blur

There are various forms of motion intrinsic to cell biology. For example, the relay helix movement in kinesin structures can be described as a translational displacement [1], rotational movements can be observed in actin filaments [2] and the stochastic step-wise movement of motor proteins can be modeled as thermal Brownian motion [3].

To account for this variety in the dynamics of cell biology, we first analyze the effect of arbitrary movements on image formation. To this extent, let us consider an arbitrary moving object $o(x, y, t)$, with a corresponding time-varying spatial frequency spectrum $\mathcal{O}(f_x, f_y, t)$. Furthermore, we consider a time-invariant imaging system with point spread function (PSF) $p(x, y)$, equivalently expressed in the spatial frequency domain through the optical transfer function (OTF) $P(f_x, f_y)$. Under the assumption that the acquisition speed is sufficiently high so that the motion is captured with high fidelity, the measured image can be modeled as an integration over the acquisition time window $[t_0, t_0 + \Delta t]$. The observed image spectrum $I(f_x, f_y)$ can therefore be written as:

$$I(f_x, f_y) = \mathcal{F}_{xy}\left\{\int_{t_0}^{t_0+\Delta t} p(x, y) * o(x, y, t)\ \mathrm{d}t\right\} \tag{7}$$

$$= P(f_x, f_y) \int_{t_0}^{t_0+\Delta t} \mathcal{O}(f_x, f_y, t)\ \mathrm{d}t. \tag{8}$$

From Equation (8), we see that the measured image spectrum is a temporal accumulation of the spectrum of the moving ground-truth object, modulated by the OTF of the imaging system. As the temporal integration is not invertible, this frequency information is irretrievably lost in the final image. The only way to acquire this information is to reduce the acquisition time Δt.

To continue our analysis of Equation (8), we now specifically focus on translation and rotation as biologically relevant examples of linear motion [1, 2]. Translational motion in the spatial domain appears as a phase modulation in the frequency domain. This can be modeled as

$$\mathcal{O}(f_x, f_y, t) = e^{j(f_x \Delta x(t) + f_y \Delta y(t))}\mathcal{O}(f_x, f_y, t_0), \tag{9}$$

where $\Delta x(t)$ and $\Delta y(t)$ denote the positive displacement of the moving object coordinates along x and y directions. Substituting this into Eq. (8), the measured spectrum becomes

$$I(f_x, f_y) = P(f_x, f_y)\mathcal{O}(f_x, f_y, t_0) \int_{t_0}^{t_0+\Delta t} e^{j(f_x \Delta x(t) + f_y \Delta y(t))}\ \mathrm{d}t \tag{10}$$

The final expression for the magnitude of the measured spectrum of an object moving with constant translation motion with v_x in x direction and v_y direction can be derived starting from Equation (10) as follows:

$$|I(f_x, f_y)| = |P(f_x, f_y)|\ \left|\frac{-j}{f_x v_x + f_y v_y}\right|\ |\mathcal{O}(f_x, f_y, t_0)|\ \left|e^{j t_0 (f_x v_x + f_y v_y)}\right|\ \left|(e^{j\Delta t(f_x v_x + f_y v_y)} - 1)\right| \tag{11}$$

$$= P(f_x, f_y)\ \frac{1}{f_x v_x + f_y v_y}\ |\mathcal{O}(f_x, f_y, t_0)|\ |(e^{j\Delta t(f_x v_x + f_y v_y)} - 1)| \tag{12}$$

$$= P(f_x, f_y)\ \frac{2}{|f_x v_x + f_y v_y|}\ |\mathcal{O}(f_x, f_y, t_0)|\ |\sin\left(\frac{\Delta t(f_x v_x + f_y v_y)}{2}\right)| \tag{13}$$

$$= P(f_x, f_y)\ |\mathcal{O}(f_x, f_y, t_0)| \frac{|\sin\left(\frac{\Delta t(f_x v_x + f_y v_y)}{2}\right)|}{\frac{|f_x v_x + f_y v_y|}{2}} \tag{14}$$

$$= P(f_x, f_y)\, \Delta t \left|\mathcal{O}(f_x, f_y, t_0)\, \mathrm{sinc}\left(\frac{\Delta t(f_x v_x + f_y v_y)}{2}\right)\right|. \tag{15}$$

As shown in Equation (15), translational motion introduces a sinc modulation in the frequency domain, where the degree of attenuation depends on the projection of the spatial frequency vector onto the velocity vector (v_x, v_y). Thus, frequency components aligned with the motion are preferentially suppressed, reducing edge sharpness in that direction, while components orthogonal to the motion are less affected and appear relatively enhanced. For example, when a vertical line translates along the $+x$ direction (see Figure 3), edges parallel to the y-axis become blurred, reflecting a loss of horizontal frequency components and a relative strengthening of vertical ones. In this way, varying exposure time not only redistributes spectral energy via suppression by sinc modulation and scaling by the duration of the exposure time, but also biases the visibility of frequency orientations, producing anisotropy in the spectrum. Additionally, the exposure time Δt acts as a proportional factor for both intensity and spectral scaling: longer Δt increases photon collection but narrows the effective spectral bandwidth defined by the sinc main lobe.

We do an analogous derivation for rotational motion. Let us consider a counter-clockwise rotation at a constant angular velocity ω. In polar coordinates (ρ, ϕ), spectrum at time t relates to the initial spectrum at $t = t_0$ as

$$\mathcal{O}(\rho, \phi, t) = \mathcal{O}_0(\rho, \phi - \omega t). \tag{16}$$

The measured spectrum therefore becomes

$$I(\rho, \phi) = P(\rho, \phi) \int_{t_0}^{t_0+\Delta t} \mathcal{O}_0(\rho, \phi - \omega t)\, \mathrm{d}t. \tag{17}$$

Substituting $\theta = \omega t$ with $\mathrm{d}\theta = \omega\, \mathrm{d}t$, this simplifies to

$$I(\rho, \phi) = \frac{P(\rho, \phi)}{\omega} \int_{\omega t_0}^{\omega(t_0+\Delta t)} \mathcal{O}_0(\rho, \phi - \theta)\, \mathrm{d}\theta. \tag{18}$$

Equation (18) demonstrates that rotational motion at a constant angular velocity results in a radial averaging of the original spectrum. Unlike translation, which induces anisotropic suppression of frequencies aligned with the motion direction, rotation homogenizes the angular structure of the spectrum. As a consequence, radial frequency content can appear well-preserved, while angular information carrying critical structural signatures is progressively lost.

3 Simulation

In this section, we describe the simulation tool used in this paper. The simulation pipeline consists of four steps: the definition of the object and its movement in continuous space and time, the discretization of the object onto a fine spatio-temporal mesh, spatial convolution with a point spread function to arrive at a band-limited object representation, and a simulation of the camera readout process. Moreover, we list the simulation parameters and the default values used in our simulations.

3.1 Object formation

We simulated two representative object types under controlled motion: a rectangular structure undergoing translation and a Siemens star undergoing rotation. The line was modeled as a thin rectangular object, which, in the absence of motion, ideally exhibits a single dominant frequency component orthogonal to its main axis (Supplementary Figure 2), excluding the influence of the PSF. This property makes it particularly suitable for isolating and analyzing the effects of translational motion. The Siemens star, a well-established standard for spatial resolution assessment [4], was selected to examine rotational motion. Owing to its radially symmetric design, rotation of the Siemens star induces a uniform angular displacement of its spatial frequency components across all radii, providing an ideal framework for evaluating motion effects in the rotational domain.

3.1.1 Object-independent transformations

- **Input**: sampled timepoints t, sampled simulation space coordinates x, y.
- **Motion parameters** $\vartheta_{\mathrm{motion}}$: initial object center coordinates x_0, y_0, translation velocities v_x, v_y, initial angle ϕ_0, rotation period P.

First, we apply *translation* to the initial center coordinates of the object.

$$x_c(t) = x_0 + v_x t, \tag{19}$$
$$y_c(t) = y_0 + v_y t \tag{20}$$

Next, create a local coordinate system centered around the object center by applying

$$x_{local}(t) = x - x_c(t), \tag{21}$$
$$y_{local}(t) = y - y_c(t) \tag{22}$$

Next, we apply *rotation* to the local coordinates by calculating the inclination angle:

$$\phi(t) = \phi_0 + \frac{2\pi}{P} t \tag{23}$$

Therefore, the rotation operations $\mathcal{R}_x(x, x_c(t), \phi(t))$ in the x-axis and $\mathcal{R}_x(y, y_c(t), \phi(t))$ in the y-axis are defined such that the sampling coordinates after the rotation results in:

$$\mathcal{R}_x(x, x_c(t), \phi(t)) = x_r(t) = x_{local}(t)\cos(\phi(t)) - y_{local}(t)\sin(\phi(t)), \tag{24}$$
$$\mathcal{R}_y(y, y_c(t), \phi(t)) = y_r(t) = x_{local}(t)\sin(\phi(t)) + y_{local}(t)\cos(\phi(t)) \tag{25}$$

3.1.2 Object example: Line

- **Object parameters** $\vartheta_{\mathrm{object}}$: height H, width W.
- **All parameters**: $\vartheta = \vartheta_{\mathrm{motion}} \cup \vartheta_{\mathrm{object}}$.

The line object is defined as a rectangle of height H and width W. The objects in our simulation are defined with respect to the time-dependent rotated coordinate system with the object center being its origin. Therefore, the object area/mask $\mathcal{A}(t, \vartheta)$ at a timepoint t given all parameters ϑ is defined as following:

$$\mathcal{A}(t, \vartheta) = \{(x, y) : (|\mathcal{R}_x(x, x_c(t), \phi(t))| \leq \frac{W}{2} + \varepsilon) \cap (|\mathcal{R}_y(y, y_c(t), \phi(t))| \leq \frac{H}{2} + \varepsilon)\} \tag{26}$$

where we add $\varepsilon \approx 10^{-14}$ for numerical stability.

3.1.3 Object example: Siemens Star

- **Object parameters** $\vartheta_{\mathrm{object}}$: number of spokes N_s, maximum radius $r_{\max}$.
- **All parameters**: $\vartheta = \vartheta_{\mathrm{motion}} \cup \vartheta_{\mathrm{object}}$.

The Siemens star object [4] is comprised of N_s sinusoidal waves in a polar coordinate system $(r(t), \Theta(t))$. We can convert the rotated Cartesian coordinates to polar by:

$$r(t) = \sqrt{x_{\mathrm{r}}^2(t) + y_{\mathrm{r}}^2(t)}, \tag{27}$$
$$\Theta(t) = \arctan\left(\frac{y_{\mathrm{r}}(t)}{x_{\mathrm{r}}(t)}\right) \tag{28}$$

In its general form, it is defined as follows:

$$\mathcal{A}(\Theta(t)) = \mathcal{P}_a + \mathcal{P}_b \sin(N_s\Theta(t) - \varphi), \tag{29}$$

where $\mathcal{P}_a$ represents mean pattern intensity value, $\mathcal{P}_b$ - the amplitude of the oscillations, and φ is the optional phase offset. In our simulations, we choose $\mathcal{P}_a = 0$, $\mathcal{P}_b = 1$. Additionally, we choose $\varphi = \frac{\pi}{2}$ in order to have the Siemens star at peak intensity for the $\Theta(t) = 0$. Therefore, our Siemens star definition simplifies to:

$$\mathcal{A}(\Theta(t)) = \cos(N_s\Theta(t)), \tag{30}$$

Furthermore, we restrict the maximum radius of the Siemens star if needed, and obtain the binary definition of the Siemens star $\mathcal{A}(t, \vartheta)$ at a timepoint t given all parameters ϑ as follows:

$$\mathcal{A}(t, \vartheta) = \{(x, y) : (\cos(N_s\Theta(t)) > 0) \cap (r(t) \leq r_{\max})\} \tag{31}$$

3.1.4 Object example: Point with Brownian motion

- **Object parameters** $\vartheta_{\text{object}}$: diffusion coefficient D.
- **All parameters**: $\vartheta = \vartheta_{\text{motion}} \cup \vartheta_{\text{object}}$.

Note that we do not include rotation in the motion model for this object. The object is defined at its local coordinate system as described above at center coordinates $x_{local}(t)$, $y_{local}(t)$. Then, we apply Brownian motion to the point object, thus altering the center coordinates as follows:

$$\mathcal{B}(x_{local}(t), D) = x_{local}(t) + \xi_x(D), \tag{32}$$

$$\mathcal{B}(y_{local}(t), D) = y_{local}(t) + \xi_y(D), \tag{33}$$

where $\xi_x, \xi_y \sim \mathcal{N}(0, 2D\Delta t)$ and $\Delta t = t_i - t_{i-1}$.

Then, the object mask $\mathcal{A}(t, \vartheta)$ for the point object on the sampled coordinate grid x, y is defined as follows:

$$\mathcal{A}(t, \vartheta) = \{(x, y) : (|\mathcal{B}(x_{local}(t), D) - \frac{\Delta x}{2}| \leq 0) \cap (|\mathcal{B}(y_{local}(t), D) - \frac{\Delta y}{2}| \leq 0)\}, \tag{34}$$

where $\Delta x = x_i - x_{i-1}$, $\Delta y = y_i - y_{i-1}$.

3.2 Definition of object and movement

We consider an object $o(x, y, t)$ at time t. The shape of the object is defined by $S(x, y, t)$. We limit ourselves to binary object shapes: $S(x, y, t)$ takes value 1 if the coordinates (x, y) are within an arbitrarily-shaped time-dependent object area $\mathcal{A}(t)$ centered at spatial coordinates $(x(t), y(t))$ at time t, and 0 otherwise:

$$S(x, y, t) = \begin{cases} 1, \text{ if } (x, y) \in \mathcal{A}(t), \\ 0, \text{ otherwise.} \end{cases} \tag{35}$$

To convert the shape into an object, we introduce θ_I, which represents the expected number of signal photons per unit area (nm^2) and per unit time (ms), and θ_{bg}, which represents the expected number of background photons per unit area per unit time. This gives the following definition of the object:

$$o(x, y, t) = \theta_I S(x, y, t) + \theta_{bg}. \tag{36}$$

This object model is a simplified version of that in Section 2, with the expected intensity set to a uniform value.

3.3 Discrete representation of ground truth object

To be able to represent the continuous-space, continuous-time ground truth object as a simulated video, the object is discretized on a fine mesh in space and time. For the discretization in space, we define the mesh widths δx, δy, and the number of mesh pixels n_x, n_y. We align the continuous-space coordinate system with the discrete-space mesh, such that the center of the discrete-space mesh aligns with the continuous-space origin coordinates $(0,0)$. For each mesh pixel with indices (i,j), where $i \in \{0,1,\cdots,n_x-1\}$ and $j \in \{0,1,\cdots,n_y-1\}$, we have that its center coordinates are $(x^{(i)}, y^{(j)})$ with $x^{(i)} = i\delta x - \frac{(n_x-1)\delta x}{2}$ and $y^{(j)} = j\delta y - \frac{(n_y-1)\delta y}{2}$.

For the discretization in time, we define the sampling interval δt, the number of time samples n_t, and the time index $k \in \{0,1,\cdots,n_t-1\}$, such that at each time step k, $t^{(k)} = k\delta t$.

Using this discretization, we now map the object and its motion to discrete space and time domain. In the discrete-space mesh, pixel (i,j) of the discrete-space object takes the value of the continuous-space object if the center coordinates of the mesh pixel lie within the continuous-space object at time step k. Additionally, the object definition flux $o(x,y,t)$ is adjusted to account for the mesh voxel size $\delta V = \delta x \cdot \delta y \cdot \delta t$ ($\mathrm{nm}^2 \cdot \mathrm{ms}$). This results in the following expression for the discrete-space object $o_{i,j,k}$:

$$o_{i,j,k} = o(x^{(i)}, y^{(j)}, t^{(k)})\ \delta V. \tag{37}$$

3.4 Ground truth image formation

During the image formation process, the imaging system distorts an object through a point spread function, which represents the accumulated effects of diffraction and any optical disturbances that occur in the imaging system. The image results from convolving the object with the point spread function, and is therefore a smoothed version of the object.

To simulate the image formation in discrete space, we define the point spread function through a blurring kernel $\mathrm{PSF}_{i,j,k}$, defined on the discrete-space, discrete-time mesh of Subsection 3.3. Similarly to the object, the point spread function can in principle be of an arbitrary shape and it can change over time. We choose to represent the point spread function via a time-invariant two-dimensional Gaussian kernel $\mathrm{PSF}_{i,j}(\sigma,s) = G_{i,j}(\sigma,s)$. Since the two-dimensional Gaussian kernel is separable in x and y, we have $G_{i,j}(\sigma,s) = G_i(\sigma,s)G_j(\sigma,s)$. Here, σ denotes the standard deviation and s is the kernel size, which has to be an odd number in pixels for symmetric discretization of the Gaussian PSF.

It is important to note that the PSF convolution step takes place in the continuous space domain and is then sampled via discretization in all three spatio-temporal dimensions. Hence, the conditions for which the expression in Equation (38) is an accurate approximation of this operation should be clearly stated. We assume that

- The object can be approximated as band-limited.
- The Gaussian PSF can be approximated as a band-limited function of space.
- The object and the PSF are sampled at frequencies higher than the Nyquist rate.

In a simulation environment, we can freely choose s and δx such that they provide accurate approximations to continuous convolution. After satisfying the Nyquist sampling condition, the convolution in simulation space was performed over a field-of-view area of at least 6.7 σ_{PSF} to ensure negligible truncation artifacts.

The ground truth image $I_{i,j,k}$ in the image plane, before the camera records it, is now given by the following two-dimensional convolution in space (over the i,j coordinates), for each time step k:

$$I_k = o_k * \mathrm{PSF}(\sigma, s). \tag{38}$$

3.5 Simulation of measurements

During a measurement, a camera converts a continuous-space, continuous-time moving image into a sequence of discretized measurements. Specifically, the image is recorded by an array of camera pixels, resulting in coarse discretizations in space. Furthermore, each measurement takes a finite amount of time, resulting in coarse discretizations in time as well. During the acquisition, the signal coming from the moving image gets accumulated, resulting in motion blur.

For the discretization in space and time by the camera, we describe the camera pixel size as Δx, Δy obtained with the exposure time Δt, and the number of camera pixels as N_x, N_y with the number of camera frames as N_t. These can be described as coarse discretized voxels with indices (p, q, r), where $p \in \{0, \cdots, N_x - 1\}$, $q \in \{0, \cdots, N_y - 1\}$, $r \in \{0, \cdots, N_t - 1\}$, and should line up with the coordinate system of the finely discretized object and image, introduced in Subsection 3.3.

To ensure this condition, we require that the number of pixels n_x, n_y, and temporal samples n_t used to represent the finely discretized object and image be integer multiples of the corresponding numbers on the coarser measurement grid, denoted by N_x, N_y, and N_t. Specifically, $n_x = C_x N_x$ along the x-direction, $n_y = C_y N_y$ along the y-direction, and $n_t = C_t N_t$ for temporal sampling, where C_x, C_y, and C_t are integers.

From the relations above, the pixel sizes of the fine object and image grids are determined by those of the coarse measurement grid as $\delta x = \frac{\Delta x}{C_x}$, $\delta y = \frac{\Delta y}{C_y}$, and $\delta t = \frac{\Delta t}{C_t}$. In practice, the camera pixel size and exposure time are fixed by the experimental setup, while the choice of C_x, C_y, and C_t fully specifies the finer grid on which the ground-truth object and image are represented. This grid should satisfy the spatial and temporal Nyquist criteria, ensuring that the highest spatial and temporal frequencies of the continuous-domain ground truth are sampled at least twice per period. To capture all relevant details faithfully, however, we recommend a denser sampling, typically oversampling the maximum spatial and temporal frequencies by a factor of ten.

As the coarse measurement grid and the fine discrete-space representation of the image now share a coordinate system and overlap, the mapping between these grids is straightforward. On each camera voxel (p, q, r), the intensity is represented by summing all the pixels of the fine image grid that lie within the pixel area of the coarse grid, as well as all the time samples of the fine image grid that lie within the camera frame duration. The discretized image $\hat{I}$ can be represented as follows:

$$\hat{I}_{p,q,r} = \sum_{i=0}^{C_x-1} \sum_{j=0}^{C_y-1} \sum_{k=0}^{C_t-1} I_{pC_x+i, qC_y+j, rC_t+k}. \tag{39}$$

3.5.1 Noise models

Finally, we simulate shot noise by sampling from Poisson distribution with $I_{p,q,r}$ being the distribution parameter:

$$\hat{I}^{\text{noised}}_{p,q,r} \sim \text{Pois}(\hat{I}_{p,q,r}). \tag{40}$$

As independent Poisson processes with rates $\{\lambda_i\}$ merge into a single process of rate $\sum_i \lambda_i$, the total accumulated signal needs only to be sampled once.

3.6 Biological relevance for chosen velocities

Table 1 describes all default values set for the simulation parameters throughout our work. For the biologically-relevant parameters, the following works have been found relevant.

Nanostructures like kinesin can develop a directed (averaged) speed of 0.8 nm/ms (800 nm/s) [8]. Alternatively, proteins of around 5 nm radius can exhibit a Brownian motion with a diffusion coefficient around 15 $\mu m^2/s$.

Motions of larger objects like bacterial or whole-cell motility can reach considerably higher translational/directed motion, ranging from 8 to 50 nm/ms. For instance, Karim et al. [7] made measurements of motility of three gastrointestinal pathogens, Helicobacter pylori, Campylobacter jejuni, and Escherichia coli. The authors state: "C jejuni moved fastest, with a median curvilinear velocity (CLV) of 38.76 microns/s (range 29.08 to 52.82). Next was H pylori, median CLV 25.02 microns/s (range 12.07 to 29.07). E coli was the slowest, median CLV 12.73 microns/s (range 8.20 to 18.04)." The

Table 1: Default simulation parameters used throughout our work

**Parameter**	**Value**	**References**
**Object parameters**		
Center coordinates in x- and y-axes	(0, 0)	
Initial angle	0	
Signal photon flux	$5 \cdot 10^{-4} \frac{\text{photons}}{nm^2 \cdot ms}$	
Background photon flux	$5 \cdot 10^{-5} \frac{\text{photons}}{nm^2 \cdot ms}$	
Line velocity in x-axis	40 nm/ms	
Line velocity in y-axis	0 nm/ms	
Line height	3600 nm	
Line width	72 nm	
Siemens star number of spokes N_s	8 or 3	
Siemens star maximum radius $r_{\max}$	3600 nm or ∞ (unlimited)	
**PSF-related parameters**		
Emission wavelength λ	690 nm	[5]
Numerical aperture (NA)	1.4	[5]
Standard deviation of Gaussian	$0.21\lambda/\text{NA} \approx 103.5nm$	[6]
Gaussian kernel size	360 pixels (= FOV)	
**Simulation space parameters**		
Mesh pixel width δx	20 nm	
Mesh pixel height δy	20 nm	
Mesh pixel number n_x	360	
Mesh pixel number n_y	360	
Time sampling interval δt	1 ms	
Time sampling number n_t	120	
**Camera parameters**		
Pixel size Δx	60 nm	[5]
Pixel size Δy	60 nm	[5]
Pixel count N_x	120	
Pixel count N_y	120	
Camera exposure time	40 ms	
**Motion and intensity parameters**		
Object translation velocity	1-50 nm/ms	[7, 8]
Period of rotation	1000-3000 ms/rotation (0.3-1 rot/s (Hz), 20-60 rot/min)	[2],[9, 10, 11]
Diffusion coefficient	3.5-15 $\mu m^2/s$	[12]
Signal photon flux	$2.5 \cdot 10^{-5} - 5 \cdot 10^{-3} \frac{\text{photons}}{nm^2 \cdot ms} (10 - 2000 \frac{\text{photons}}{\text{pixel} \cdot \text{frame}})$	Zeiss manual
Background photon flux	$0 - 5 \cdot 10^{-5} \frac{\text{photons}}{nm^2 \cdot ms} (0 - 20 \frac{\text{photons}}{\text{pixel} \cdot \text{frame}})$	

sizes of the bacteria vary around 2-3 microns in length and 0.7-0.9 microns in diameter [13]. Motions of biological structures are not limited to translation, however. For example, pre-mitotic organelle spinning has been reported in mouse embryonic stem cells (mESCs), where multiple organelles rotate simultaneously at an average rate of approximately 5 degrees per minute [11]. At even higher rotational speeds, the bacterial flagellar motors of *S. typhimurium* and *E. coli* can reach rotation speeds of up to 300 Hz [10].

4 Estimation of spectral signal-to-noise ratio

The advent of fluorescence super-resolution microscopy [14, 15, 16] has extended the achievable spatial resolution beyond the diffraction limit. As a result, rather than being solely based on optical parameters, resolution is often measured based on the information content of the acquired images, and thus includes the camera properties in the resolution estimation. A widely adopted image-based resolution metric is Fourier Ring Correlation (FRC) [17], which calculates the cross-correlation between two independent image realizations and defines a resolution cut-off at a fixed threshold. Similarly, decorrelation analysis (DA) estimates the highest self-consistent spatial frequency within a single image by measuring correlations across band-pass filtered versions of it [18]. Alternatively, a metric that directly quantifies the image quality from the image spectrum is the spectral signal-to-noise ratio (SSNR) [19]. Similar to its spatial-domain counterpart, the signal-to-noise ratio (SNR), SSNR measures the ratio of signal power to the power of noise across the frequency spectrum of the image. To this end, we opted for SSNR as the resolution metric. Unlike FRC and DA, which are correlation-based and normalized, SSNR is derived directly from the image spectrum and scales with photon accumulation. This makes it a suitable candidate to investigate the degradation of the spatial resolution under the effect of motion blur.

A central step in our analysis is the estimation of the spectral signal-to-noise ratio (SSNR), which quantifies the balance between structured signal and stochastic noise across spatial frequencies. In the Poisson noise regime considered here (see Equation (5)), this balance depends jointly on object and background photon rates. One of the possible approaches to find the noise power is by analytically estimating the signal and the noise power if the background photon rate is known, typically using variance-stabilizing transformations [20, 21]. However, this approach relies on the accuracy of the background model and hence may not always capture the conditions during the acquisition. Instead, we employ a procedure that infers the signal and noise power directly from batches of noisy image realizations. This bases SSNR estimates on the measured image statistics rather than on assumed acquisition parameters or purely analytical estimation, making our approach more practically relevant.

We simulate one noise-free frame $\hat{I}_{p,q,0}$ using Equation (4) and generate $N_s \times N_b$ noisy realizations (N_s samples for each of N_b batches) according to a Poisson distribution with locally expected value of $\hat{I}_{p,q,0}$. For each realization, we compute the Fourier transform $\mathcal{F}^{(i,b)}(u,v)$, with (u,v) denoting discrete Cartesian frequency coordinates and (i,b) representing the ith sample from the bth batch. The SSNR map for a batch is then estimated as

$$\mathrm{SSNR}^{(b)}(u,v) = \frac{\left|\overline{\mathcal{F}}^{(b)}(u,v)\right|^2}{\frac{1}{N_s}\sum_{i=0}^{N_s-1}\left|\mathcal{F}^{(i,b)}(u,v) - \overline{\mathcal{F}}^{(b)}(u,v)\right|^2}, \tag{41}$$

where $\overline{\mathcal{F}}^{(b)}(u,v)$ denotes the average spectrum across the N_s realizations for batch b. The numerator of Equation (41) captures the coherent signal across samples, while the denominator reflects the variability across realizations, namely the noise power.

Finally, we repeat the estimation across N_b batches to reduce the sensitivity to a single set of Poisson draws and compute the averaged Cartesian pixel maps $\mathrm{SSNR}(u,v)$ as follows:

$$\mathrm{SSNR}(u,v) = \frac{1}{N_b}\sum_{b=0}^{N_b-1}\mathrm{SSNR}^{(b)}(u,v). \tag{42}$$

To obtain a direction-independent resolution metric and follow common practice in methods such as FRC and DA, we express the Cartesian maps in Equation (42) in polar coordinates and compute the radial average $\langle\mathrm{SSNR}\rangle(\rho)$ by averaging over the angular spectral variable ϕ as follows:

$$\mathrm{SSNR}(u,v) \xrightarrow{\text{polar transform}} \mathrm{SSNR}(\rho,\phi), \tag{43}$$

$$\langle\mathrm{SSNR}\rangle(\rho) = \frac{1}{2\pi\rho}\int_{\phi} \mathrm{SSNR}(\rho,\phi)\,d\phi. \tag{44}$$

In the Results section, we report the estimated Cartesian SSNR maps and their radial averages with the associated precisions. The latter eliminates angular dependency, focusing the analysis solely on the dependence on the radial frequency ρ.

5 Background SSNR calculation

For the background level $\mathrm{SSNR}_{\mathrm{bg}}(\rho)$, we calculate $\mu_{\mathrm{bg}}(\rho)$ and σ_{bg} the following way. For obtaining $\mu_{\mathrm{bg}}(\rho)$, we first apply a polar transform to each sample in the batch:

$$\mathrm{SSNR}^{(b)}(u,v) \xrightarrow{\text{polar transform}} \mathrm{SSNR}^{(b)}(\rho,\phi). \tag{45}$$

Then, we obtain the radial averages of each SSNR estimation using the expression given by Equation (44):

$$\langle\mathrm{SSNR}\rangle^{(b)}(\rho) = \frac{1}{2\pi\rho}\int_{\phi} \mathrm{SSNR}^{(b)}(\rho,\phi)\,d\phi. \tag{46}$$

And finally, we calculate the mean across the realizations in the given batch:

$$\mu_{\mathrm{bg}}(\rho) = \frac{1}{N_b}\sum_{b=0}^{N_b-1} \langle\mathrm{SSNR}\rangle^{(b)}(\rho). \tag{47}$$

For the estimation precision σ_{bg}, we follow the same procedure in Equations (45) and (46) and we calculate the standard deviation of $\mathrm{SSNR}^{(b)}(\rho,\phi)$ across the realizations in the given batch.

6 Estimation of resolution

To quantify the resolution from the $\langle\mathrm{SSNR}\rangle(\rho)$ curve, a cut-off frequency, representing the largest spatial frequency of the image, should be defined. Other metrics, like FRC, employ various fixed thresholds, e.g. 1/2 [22, 23] and 1/7 [24]. Since SSNR has an approximate relationship with FRC [25] as $\mathrm{FRC} \approx \frac{\langle\mathrm{SSNR}\rangle}{1+\langle\mathrm{SSNR}\rangle}$, a straightforward approach would be to obtain the cut-offs from this relationship. This could be done either by calculating the FRC curve and applying the threshold to obtain the cut-off frequency, or by converting the FRC threshold into the SSNR threshold and applying it to the $\langle\mathrm{SSNR}\rangle(\rho)$ curve. However, we demonstrate in Supplementary Section 7 that the frequencies at the threshold values obtained via this approximate relationship between FRC and SSNR do not match. This makes adopting the FRC thresholds for $\langle\mathrm{SSNR}\rangle$ unsuitable.

Therefore, we opt for an SSNR-based cut-off. To determine the SSNR level where the object contribution to the signal power is zero, we introduce the SSNR of only the background $\mathrm{SSNR}_{\mathrm{bg}}(\rho)$. It incorporates the mean of radial averages of the background SSNR across realizations in a batch (see Supplementary Section 5), which we define as $\mu_{\mathrm{bg}}(\rho)$, and the estimation precision of the background SSNR $\sigma_{\mathrm{bg}}(\rho)$ calculated as the standard deviation across batches, thus representing an uncertainty interval for the background level. The threshold is then given by:

$$\mathrm{SSNR}_{\mathrm{bg}}(\rho) = \mu_{\mathrm{bg}}(\rho) + \sigma_{\mathrm{bg}}(\rho), \tag{48}$$

where $\mu_{\mathrm{bg}}(\rho)$ is the batch-wise mean of radial-averaged SSNR pixel maps, and $\sigma_{\mathrm{bg}}(\rho)$ is given by the standard deviation of radial average estimates of different batches. In line with our simulation framework, we assume independence between background and object intensities. Thus, we perform

an object-free simulation with the same background photon flux as in the main experiments. By calculating $\mathrm{SSNR_{bg}}(\rho)$ based on the background levels, we ensure that this baseline serves as a dataset-specific benchmark for assessing motion-induced resolution limits.

Following the computation of this background-only noise level in Equation (48), we propose a cut-off defined by the intersection between the $\langle\mathrm{SSNR}\rangle$ and $\mathrm{SSNR_{bg}}(\rho)$. Specifically, we define the frequency cut-off ρ_c as the largest radial frequency ρ at which the averaged SSNR remains one standard deviation above the mean background level:

$$\rho_c = \max\{\rho : \langle\mathrm{SSNR}\rangle(\rho) \ \geq \ \mathrm{SSNR_{bg}}(\rho)\}. \tag{49}$$

Additionally, we quantify the total $\langle\mathrm{SSNR}\rangle(\rho)$ at a given spectral region to compare how much its SSNR changes over various exposure times. To this end, we evaluate the area under the $\langle\mathrm{SSNR}\rangle(\rho)$ curve (AUC) between spatial frequencies ρ_1 and ρ_2 to measure the total SSNR on that interval:

$$\mathrm{AUC}(\rho_1, \rho_2) = \int_{\rho_1}^{\rho_2} \langle\mathrm{SSNR}\rangle(\rho) d\rho. \tag{50}$$

7 FRC and SSNR

Table 4 summarizes the estimated resolution distances obtained using the procedure described in Equation (49), where distances are computed individually for each batch. These values are distinct from the inverted frequency thresholds illustrated in Figure 3. Specifically, for each batch, we determine the resolution distance by inverting the cutoff frequency and scaling the normalized inverted value according to the assumed sampling frequency (pixel size) of the method. The reported averages are expressed as mean±standard deviation.

At first glance, the resolution distances derived from FRC and SSNR appear different. This discrepancy arises from the choice of SNR metric (spatial versus spectral) and the associated thresholding definition. FRC employs a fixed 1/7 threshold, corresponding to an SNR of 50%, while our approach is based on the background SSNR level, which adapts to specific image conditions rather than enforcing a universal cutoff [25]. Yet despite this methodological distinction, both metrics consistently reveal the same trend with respect to exposure time: when the line object is not aligned with the motion, longer exposures increase the resolution distance, signaling reduced resolvability. Conversely, when the main spatial component of the object (the major axis of the thin rectangle in our simulations) is aligned with the motion, the resolution distance decreases and resolvability improves. In this case, the motion sharpens object edges in the spatial domain, resulting in constructive spectral summation during the movement. The estimation of resolution distance via FRC becomes increasingly "certain", indicated by decreasing estimation precision (standard deviation) with longer exposure times only for the line oriented at 90°; this trend is not consistently observed in any of the other cases.

Table 2: Total area under the SSNR curve for a fixed line object in Figure 2 by angle and exposure time, from 0 to the cutoff frequency. The cutoff frequency ($\mu\mathrm{m}^{-1}$) is reported per measurement.

Angle (°)	Exposure (ms)	AUC(0,ρ_c)	Cutoff frequency ($\mu\mathrm{m}^{-1}$)
0	10	1369.78	3.85
	50	6914.01	4.30
	100	13530.98	4.45
45	10	1434.88	3.87
	50	7380.92	4.32
	100	14549.53	4.49
90	10	1376.96	3.87
	50	6574.61	4.30
	100	13686.21	4.47

Table 3: Resolvable distances (d_c) and frequencies (ρ_c) estimated by two FRC schemes for the moving line in Figure 3.

Exposure (ms)	Angle (°)	d_c (nm)		ρ_c (μm^{-1})	
		2FRC	1FRC	2FRC	1FRC
10	0	274.10 ± 9.98	299.04 ± 12.66	1.29 ± 0.05	1.18 ± 0.05
	45	237.91 ± 8.26	259.35 ± 12.01	1.49 ± 0.05	1.37 ± 0.06
	90	195.23 ± 6.57	216.84 ± 9.26	1.81 ± 0.06	1.63 ± 0.07
50	0	363.92 ± 26.42	424.83 ± 33.31	0.98 ± 0.07	0.84 ± 0.07
	45	328.99 ± 21.41	373.78 ± 22.19	1.08 ± 0.07	0.95 ± 0.06
	90	160.96 ± 3.56	173.52 ± 4.60	2.20 ± 0.05	2.04 ± 0.06
100	0	396.26 ± 26.14	459.56 ± 38.49	0.90 ± 0.06	0.78 ± 0.07
	45	348.77 ± 20.63	397.41 ± 27.76	1.02 ± 0.06	0.89 ± 0.07
	90	152.89 ± 3.32	163.56 ± 4.09	2.31 ± 0.05	2.16 ± 0.05

Table 4: SSNR-based resolvable distances (d_c) and frequency thresholds (ρ_c) for three thresholds (1, 1/6, and background) for the moving line in Figure 3.

Exposure (ms)	Angle (°)	d_c (nm)			ρ_c (μm^{-1})		
		$SSNR_1$	$SSNR_{1/6}$	$SSNR_{bg}(\rho)$	$SSNR_1$	$SSNR_{1/6}$	$SSNR_{bg}(\rho)$
10	0	601.45 ± 4.09	461.43 ± 1.98	362.51 ± 1.49	1.66 ± 0.01	2.17 ± 0.01	2.76 ± 0.01
	45	543.32 ± 0.00	411.91 ± 1.92	310.80 ± 2.28	1.84 ± 0.00	2.43 ± 0.01	3.22 ± 0.02
	90	487.49 ± 4.12	354.54 ± 1.42	260.29 ± 1.81	2.05 ± 0.02	2.82 ± 0.01	3.84 ± 0.03
50	0	711.07 ± 0.00	595.51 ± 3.26	383.84 ± 4.82	1.41 ± 0.00	1.68 ± 0.01	2.61 ± 0.03
	45	616.79 ± 4.30	521.08 ± 2.53	330.33 ± 2.57	1.62 ± 0.01	1.92 ± 0.01	3.03 ± 0.02
	90	414.74 ± 1.95	319.84 ± 1.77	244.99 ± 1.68	2.41 ± 0.01	3.13 ± 0.02	4.08 ± 0.03
100	0	805.86 ± 0.00	609.02 ± 4.19	387.51 ± 3.42	1.24 ± 0.00	1.64 ± 0.01	2.58 ± 0.02
	45	717.44 ± 5.81	521.08 ± 2.53	361.45 ± 3.53	1.39 ± 0.01	1.92 ± 0.01	2.77 ± 0.03
	90	374.29 ± 1.29	299.25 ± 1.02	234.53 ± 0.95	2.67 ± 0.01	3.34 ± 0.01	4.26 ± 0.02

Table 5: Object-dominated area (AUC(0,ρ_{infl})), background-dominated area (AUC(ρ_{infl},ρ_c)), their sum (Total area), and fraction of object-dominated area (O/TA, %), for the moving line in Figure 3 by angle and exposure time. Inflection frequency (ρ_{infl}) (μm^{-1}) is shown for 0° and 45° lines.

Angle (°)	Exposure (ms)	AUC(0,ρ_{infl})	AUC(ρ_{infl},ρ_c)	AUC(0,ρ_c)	$\frac{\text{AUC}(0,\rho_{\text{infl}})}{\text{AUC}(0,\rho_c)}$	ρ_{infl} (μm^{-1})
0	10	958.46	153.28	1111.74	0.86213	0.951
	50	1890.76	149.89	2040.65	0.92655	
	100	2955.79	109.62	3065.40	0.96424	
45	10	1257.37	39.37	1296.73	0.96964	1.551
	50	2557.39	13.96	2571.36	0.99457	
	100	3916.60	8.43	3925.03	0.99785	
90	10	—	—	1315.62	—	—
	50			3273.22		
	100			6190.45		

Table 6: Total SSNR of the rotating Siemens star object in Figure 5 by period and exposure time. For each configuration, the table reports the cutoff frequency ρ_c, the AUC$(0, \rho_c)$, and its decomposition into contributions below and above $\rho = 1\mu m^{-1}$.

Period (ms)	Exposure (ms)	ρ_c (μm^{-1})	AUC(0,ρ_c)	AUC(1,ρ_c)	AUC(0, 1)
600	10	2.55	3.42×10^4	14.8	3.42×10^4
600	50	2.63	1.36×10^5	16.5	1.36×10^5
600	100	2.55	1.90×10^5	16.7	1.90×10^5
900	10	2.63	3.52×10^4	17.8	3.52×10^4
900	50	2.80	1.62×10^5	22.8	1.61×10^5
900	100	2.72	2.66×10^5	23.9	2.66×10^5
1200	10	2.63	3.39×10^4	18.4	3.39×10^4
1200	50	2.72	1.53×10^5	29.8	1.52×10^5
1200	100	2.72	2.91×10^5	31.7	2.91×10^5

Table 7: Mean angular spread of the Siemens star in Figure 5 at $\rho = 1.89$ μm^{-1} and corresponding SSNR for rotating objects of period 600, 900, and 1200, across exposure times. Each cell presents the mean angular spread $(\Delta\phi)_{avg}$ containing significant signal above background, the total normalized angular domain $(\Delta\phi)_1$ (percentage of 2π), and the mean SSNR.

Period (ms) / Exposure (ms)	600			900			1200		
	SSNR	$(\Delta\phi)_{avg}$	$(\Delta\phi)_1$	SSNR	$(\Delta\phi)_{avg}$	$(\Delta\phi)_1$	SSNR	$(\Delta\phi)_{avg}$	$(\Delta\phi)_1$
10	0.03285	0.1676	16.76	0.04276	0.1676	16.76	0.04611	0.1676	16.76
50	0.03586	0.5445	52.00	0.04613	0.4189	40.00	0.05227	0.3351	32.00
100	0.03632	6.2832	100.00	0.04628	0.7121	68.00	0.05597	0.5864	56.00

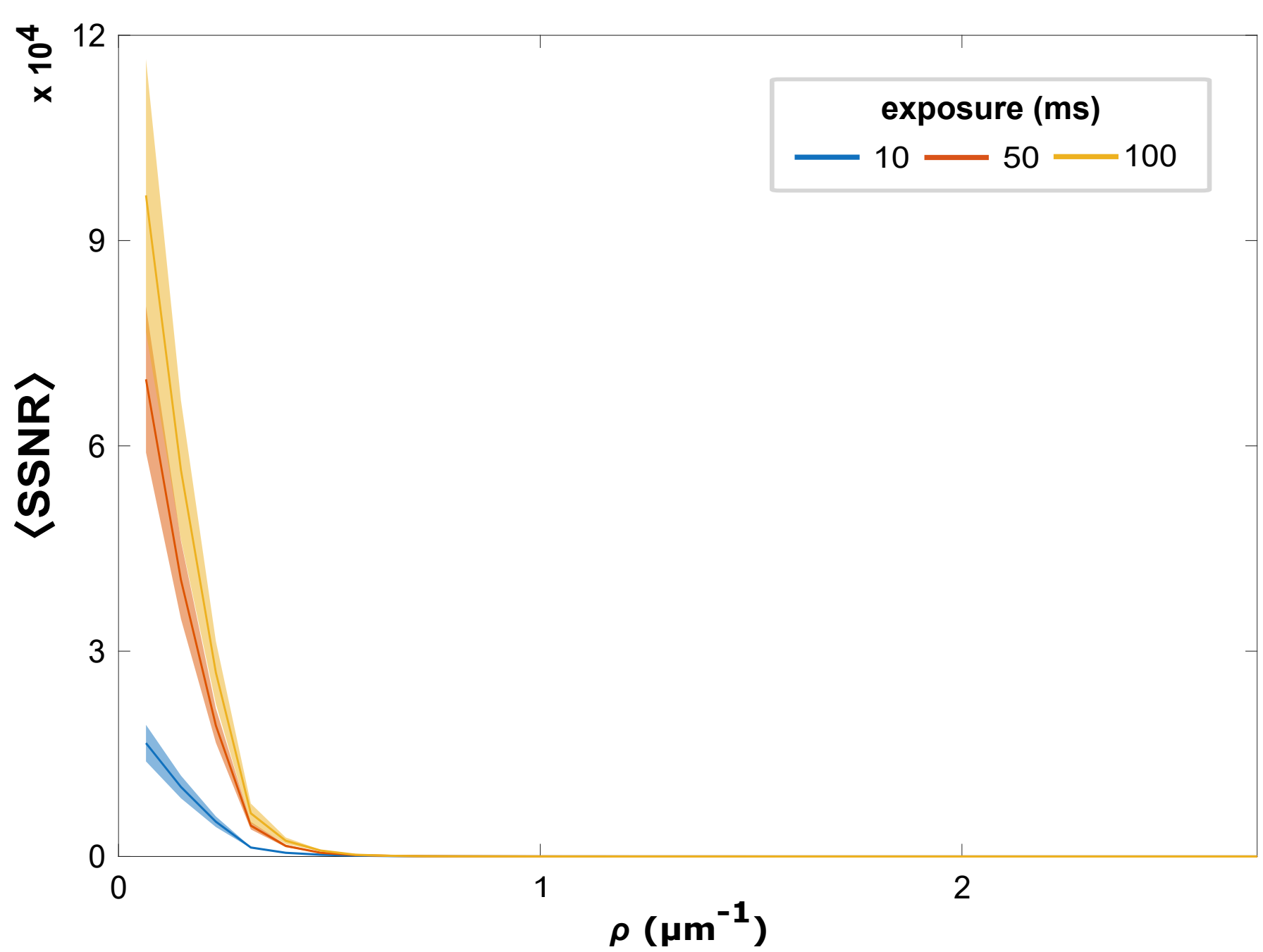

Figure 1: Linearly scaled SSNR radial averages in Figure 5.

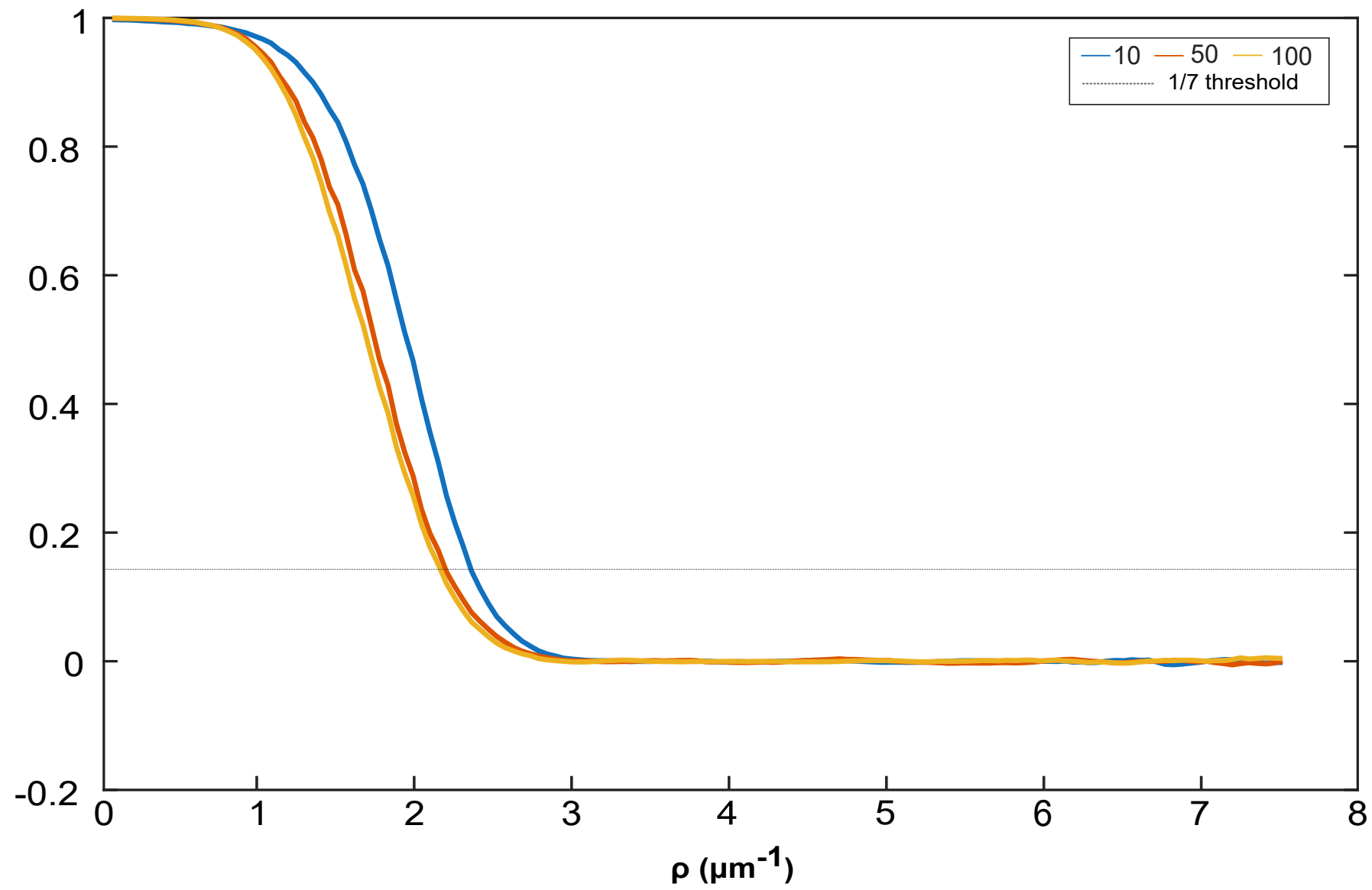

Figure 2: Averaged single image FRC curves of measurements of 4-points object in the bottom row of Figure 4*a*

Table 8: Comparison of total area under the SSNR curve (AUC(0,ρ_c)) and cutoff frequency (ρ_c) between the fixed line and moving line objects, by angle and exposure time. Moving line values use the background-threshold cutoff frequency ($SSNR_{bg}$) and the corresponding total AUC. This table compiles the information from Tables 2, 4 and 5 for a direct side-by-side comparison.

Angle (°)	Exposure (ms)	AUC(0,ρ_c)		ρ_c (μm^{-1})	
		Fixed Line	Translating Line	Fixed Line	Translating Line
0	10	1369.78	1111.74	3.85	2.76
	50	6914.01	2040.65	4.30	2.61
	100	13530.98	3065.40	4.45	2.58
45	10	1434.88	1296.73	3.87	3.22
	50	7380.92	2571.36	4.32	3.03
	100	14549.53	3925.03	4.49	2.77
90	10	1376.96	1315.62	3.87	3.84
	50	6574.61	3273.22	4.30	4.08
	100	13686.21	6190.45	4.47	4.26

8 Dynamic Drosophila data

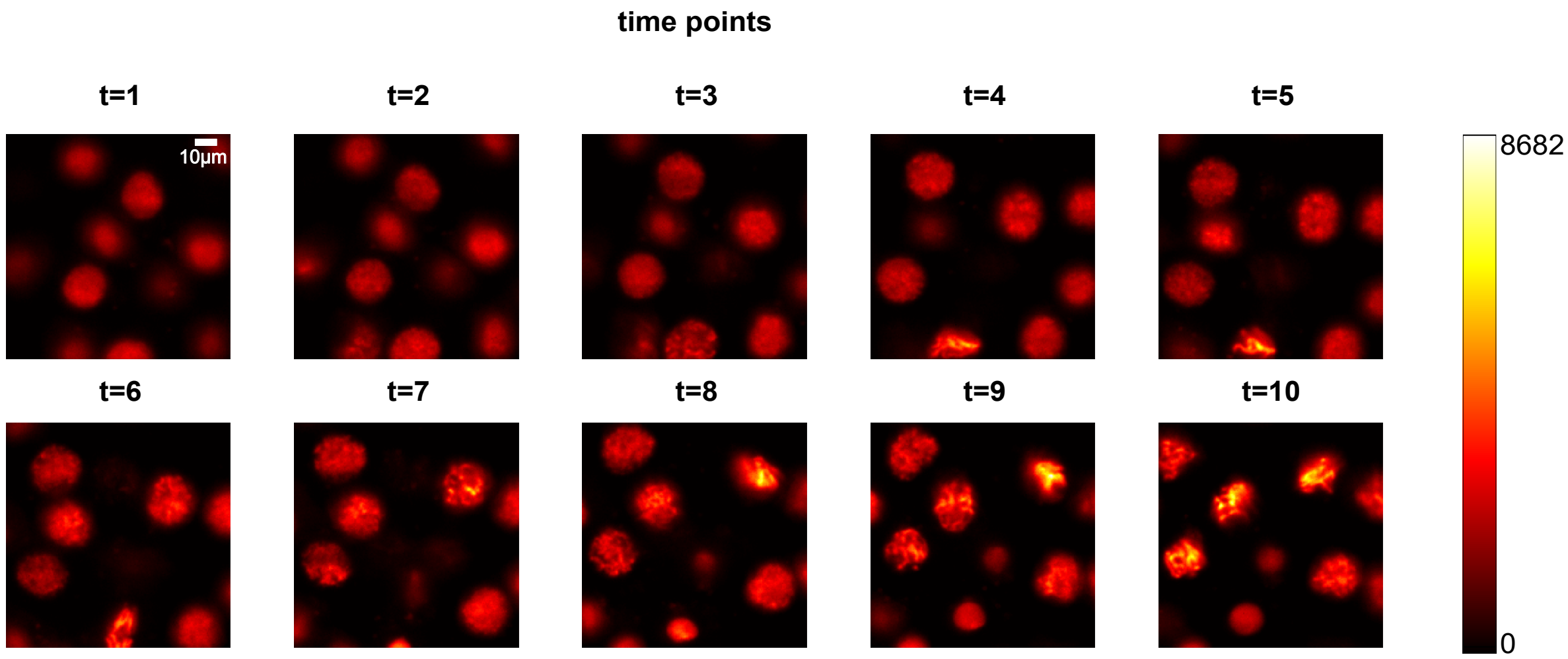

Figure 3: Time-lapse sequence used in the generation of synthetic motion blur in Figure 6
.

9 Supplementary references

References

[1] Stefan Sack et al. "X-ray structure of motor and neck domains from rat brain kinesin". In: *Biochemistry* 36.51 (1997), pp. 16155–16165.

[2] Ronald D. Vale and Ronald A. Milligan. "The Way Things Move: Looking Under the Hood of Molecular Motor Proteins". In: *Science* 288.5463 (Apr. 2000), pp. 88–95. DOI: 10.1126/science.288.5463.88.

[3] Toshio Yanagida et al. "Brownian Motion, Fluctuation and Life". In: *Biosystems*. BIOCOMP 2005: Selected Papers Presented at the International Conference - Diffusion Processes in Neurobiology and Subcellular Biology 88.3 (Apr. 2007), pp. 228–242. ISSN: 0303-2647. DOI: 10.1016/j.biosystems.2006.08.012. (Visited on 08/04/2025).

[4] Gabriel C. Birch and John C. Griffin. "Sinusoidal Siemens star spatial frequency response measurement errors due to misidentified target centers". In: *Optical Engineering* 54.7 (2015), p. 074104. DOI: 10.1117/1.OE.54.7.074104. URL: https://doi.org/10.1117/1.OE.54.7.074104.

[5] Jelmer Cnossen et al. "Localization microscopy at doubled precision with patterned illumination". In: *Nature Methods* 17.1 (Dec. 2019), pp. 59–63. DOI: 10.1038/s41592-019-0657-7.

[6] Bo Zhang, Josiane Zerubia, and Jean-Christophe Olivo-Marin. "Gaussian Approximations of Fluorescence Microscope Point-Spread Function Models". In: *Applied Optics* 46.10 (Apr. 2007), pp. 1819–1829. ISSN: 2155-3165. DOI: 10.1364/AO.46.001819.

[7] Q. N. Karim et al. "Measurement of motility of Helicobacter pylori, Campylobacter jejuni, and Escherichia coli by real time computer tracking using the Hobson BacTracker". In: *Journal of Clinical Pathology* 51.8 (Aug. 1998), pp. 623–628. DOI: 10.1136/jcp.51.8.623.

[8] David L. Coy, Michael Wagenbach, and Jonathon Howard. "Kinesin Takes One 8-nm Step for Each ATP That It Hydrolyzes". In: *Journal of Biological Chemistry* 274.6 (Feb. 1999), pp. 3667–3671. DOI: 10.1074/jbc.274.6.3667.

[9] Richard M. Berry and Howard C. Berg. "Absence of a Barrier to Backwards Rotation of the Bacterial Flagellar Motor Demonstrated with Optical Tweezers". In: *Proceedings of the National Academy of Sciences of the United States of America* 94.26 (Dec. 1997), p. 14433. DOI: 10.1073/pnas.94.26.14433.

[10] David J DeRosier. “The Turn of the Screw: The Bacterial Flagellar Motor”. In: *Cell* 93.1 (Apr. 1998), pp. 17–20. ISSN: 0092-8674. DOI: 10.1016/S0092-8674(00)81141-1.

[11] Patrick A. Sandoz et al. *Label Free 3D Analysis of Organelles in Living Cells by Refractive Index Shows Pre-Mitotic Organelle Spinning in Mammalian Stem Cells*. Sept. 2018. DOI: 10.1101/407239.

[12] Nicola Bellotto et al. “Dependence of Diffusion in Escherichia Coli Cytoplasm on Protein Size, Environmental Conditions, and Cell Growth”. In: *eLife* 11 (Dec. 2022). Ed. by Ariel Amir, Naama Barkai, and Conrad W Mullineaux, e82654. ISSN: 2050-084X. DOI: 10.7554/eLife.82654.

[13] Maira A. Constantino et al. “Helical and rod-shaped bacteria swim in helical trajectories with little additional propulsion from helical shape”. In: *Science Advances* 2.11 (Nov. 2016). DOI: 10.1126/sciadv.1601661.

[14] Stefan W. Hell and Jan Wichmann. “Breaking the diffraction resolution limit by stimulated emission: stimulated-emission-depletion fluorescence microscopy”. In: *Opt. Lett.* 19.11 (June 1994), pp. 780–782. DOI: 10.1364/OL.19.000780. URL: https://opg.optica.org/ol/abstract.cfm?URI=ol-19-11-780.

[15] M. G. L. Gustafsson. “Surpassing the lateral resolution limit by a factor of two using structured illumination microscopy”. In: *Journal of Microscopy* 198.2 (2000), pp. 82–87. DOI: https://doi.org/10.1046/j.1365-2818.2000.00710.x.

[16] Eric Betzig et al. “Imaging Intracellular Fluorescent Proteins at Nanometer Resolution”. In: *Science* 313.5793 (2006), pp. 1642–1645. DOI: 10.1126/science.1127344. eprint: https://www.science.org/doi/pdf/10.1126/science.1127344. URL: https://www.science.org/doi/abs/10.1126/science.1127344.

[17] Robert P. J. Nieuwenhuizen et al. “Measuring Image Resolution in Optical Nanoscopy”. In: *Nature Methods* 10.6 (June 2013), pp. 557–562. ISSN: 1548-7105. DOI: 10.1038/nmeth.2448.

[18] A. Descloux, K. S. Grußmayer, and A. Radenovic. “Parameter-Free Image Resolution Estimation Based on Decorrelation Analysis”. In: *Nature Methods* 16.9 (Sept. 2019), pp. 918–924. ISSN: 1548-7105. DOI: 10.1038/s41592-019-0515-7.

[19] M. Unser et al. “Spectral signal-to-noise ratio and resolution assessment of 3D reconstructions”. In: *Journal of Structural Biology* 149.3 (2005), pp. 243–255. ISSN: 1047-8477. DOI: https://doi.org/10.1016/j.jsb.2004.10.011. URL: https://www.sciencedirect.com/science/article/pii/S1047847704002047.

[20] Pakshal Bohra et al. “Variance-Stabilization-Based Compressive Inversion under Poisson or Poisson–Gaussian Noise with Analytical Bounds”. In: *Inverse Problems* 35.10 (Sept. 2019), p. 105006. ISSN: 0266-5611. DOI: 10.1088/1361-6420/ab2aa7.

[21] R. F. Wagner and D. G. Brown. “Unified SNR Analysis of Medical Imaging Systems”. In: *Physics in Medicine & Biology* 30.6 (June 1985), p. 489. ISSN: 0031-9155. DOI: 10.1088/0031-9155/30/6/001.

[22] B. Böttcher, S. A. Wynne, and R. A. Crowther. “Determination of the fold of the core protein of hepatitis B virus by electron cryomicroscopy”. In: *Nature* 386.6620 (Mar. 1997), pp. 88–91. DOI: 10.1038/386088a0.

[23] Roland Beckmann et al. “Alignment of Conduits for the Nascent Polypeptide Chain in the Ribosome-Sec61 Complex”. In: *Science* 278.5346 (1997), pp. 2123–2126. DOI: 10.1126/science.278.5346.2123. eprint: https://www.science.org/doi/pdf/10.1126/science.278.5346.2123. URL: https://www.science.org/doi/abs/10.1126/science.278.5346.2123.

[24] Peter B. Rosenthal and Richard Henderson. “Optimal Determination of Particle Orientation, Absolute Hand, and Contrast Loss in Single-particle Electron Cryomicroscopy”. In: *Journal of Molecular Biology* 333.4 (Oct. 2003), pp. 721–745. DOI: 10.1016/j.jmb.2003.07.013.

[25] Bernd Rieger et al. “Single Image Fourier Ring Correlation”. In: *Optics Express* 32.12 (June 2024), pp. 21767–21782. ISSN: 1094-4087. DOI: 10.1364/OE.524683. (Visited on 04/29/2025).
